# Physiological, morphological, and ecological tradeoffs influence vertical habitat use of deep-diving toothed-whales in the Bahamas

**DOI:** 10.1371/journal.pone.0185113

**Published:** 2017-10-11

**Authors:** Trevor W. Joyce, John W. Durban, Diane E. Claridge, Charlotte A. Dunn, Holly Fearnbach, Kim M. Parsons, Russel D. Andrews, Lisa T. Ballance

**Affiliations:** 1 Scripps Institution of Oceanography, University of California San Diego, La Jolla, California, United States of America; 2 Southwest Fisheries Science Center, National Marine Fisheries Service, National Oceanic and Atmospheric Administration, La Jolla, California, United States of America; 3 Bahamas Marine Mammal Research Organization, Marsh Harbor, Abaco, Bahamas; 4 Sea Mammal Research Unit, Scottish Oceans Institute, University of St Andrews, St Andrews, Scotland, United Kingdom; 5 SR³ SeaLife Response, Rehabilitation, and Research, Mukilteo, Washington, United States of America; 6 Marine Mammal Laboratory, Alaska Fisheries Science Center, National Marine Fisheries Service, National Oceanic and Atmospheric Administration, Seattle, Washington, United States of America; 7 School of Fisheries and Ocean Sciences, University of Alaska Fairbanks, Fairbanks, Alaska, United States of America; 8 Marine Ecology and Telemetry Research, Seabeck, Washington, United States of America; Institute of Deep-sea Science and Engineering, Chinese Academy of Sciences, CHINA

## Abstract

Dive capacity among toothed whales (suborder: Odontoceti) has been shown to generally increase with body mass in a relationship closely linked to the allometric scaling of metabolic rates. However, two odontocete species tagged in this study, the Blainville’s beaked whale *Mesoplodon densirostris* and the Cuvier’s beaked whale *Ziphius cavirostris*, confounded expectations of a simple allometric relationship, with exceptionally long (mean: 46.1 min & 65.4 min) and deep dives (mean: 1129 m & 1179 m), and comparatively small body masses (med.: 842.9 kg & 1556.7 kg). These two species also exhibited exceptionally long recovery periods between successive deep dives, or inter-deep-dive intervals (*M*. *densirostris*: med. 62 min; *Z*. *cavirostris*: med. 68 min). We examined competing hypotheses to explain observed patterns of vertical habitat use based on body mass, oxygen binding protein concentrations, and inter-deep-dive intervals in an assemblage of five sympatric toothed whales species in the Bahamas. Hypotheses were evaluated using dive data from satellite tags attached to the two beaked whales (*M*. *densirostris*, n = 12; *Z*. *cavirostris*, n = 7), as well as melon-headed whales *Peponocephala electra* (n = 13), short-finned pilot whales *Globicephala macrorhynchus* (n = 15), and sperm whales *Physeter macrocephalus* (n = 27). Body mass and myoglobin concentration together explained only 36% of the variance in maximum dive durations. The inclusion of inter-deep-dive intervals, substantially improved model fits (R^2^ = 0.92). This finding supported a hypothesis that beaked whales extend foraging dives by exceeding aerobic dive limits, with the extension of inter-deep-dive intervals corresponding to metabolism of accumulated lactic acid. This inference points to intriguing tradeoffs between body size, access to prey in different depth strata, and time allocation within dive cycles. These tradeoffs and resulting differences in habitat use have important implications for spatial distribution patterns, and relative vulnerabilities to anthropogenic impacts.

## Introduction

With a few notable exceptions, such as surface copepod aggregations exploited by *Eubaleana* spp. [1], the prey resources of cetaceans are mostly found at ocean depths ranging from tens to thousands of meters [2–5]. Due to the retention of air breathing in the secondary aquatic transition of cetaceans, accessing these deep prey resources requires commuting and limits the duration of each bout of access [6]. The vertical separation between prey resources and the surface imposes complex energetic and ecological trade-offs [7,8] that have likely played important evolutionary roles in shaping cetacean morphology and physiology. These trade-offs may have been particularly influential among lineages of specialist deep-diving toothed whales from the sub-order Odontoceti, which undertake extensive vertical travel to reach mesopelagic (200–1000 m) and bathypelagic (1000–3000 m) prey resources.

The balance of time and energy invested in commuting to a specific depth relative to the time available for prey search and acquisition at that target depth represents one of the important trade-offs for deep-diving air-breathing predators [6–10]. The amount of time different cetaceans can sustain dive apnea effectively caps the overall range of accessible dive depths based on vertical commuting velocities and two-way travel times [10,11]. Within this overall limitation, longer dives can also increase the proportion of dive time spent within a target foraging depth range relative to commuting time, thereby enabling more efficient access to prey within that depth range [6,8,9].

Different depth ranges also offer contrasting opportunities for energy and nutrient acquisition based on: 1) the heterogeneous distribution of prey density with depth [12,13] 2) variation in nutritional quality of prey with depth [14–16] 3) differences in the capacities of prey to evade capture with depth [17] and 4) vertical differences in the strength of competitive interactions with other cetacean and non-cetacean predators [18]. Given the important role of dive duration in these energetic and ecological trade-offs, we consider in this paper how cetacean morphology, physiology, and behavior interact to constrain dive durations.

Previous studies have noted a positive relationship between dive duration and body mass, specifically among the toothed whales [19], and more broadly among cetaceans, mammals, and other tetrapods [19–21]. A hypothesized mechanism underpinning this relationship proposes that both oxygen reservoirs and total oxygen demand increase as functions of body mass. However, in terms of increasing body mass, oxygen reservoirs scale approximately isometrically (*i*.*e*., linearly) while oxygen demand scales non-linearly as a power law function, based on the allometric scaling of metabolic rates [22–24]. The divergence between these two curves is hypothesized to allow larger species and individuals to sustain longer dives [20,25]. However, the added dive duration and increased energetic efficiency of a larger body mass may be counter-balanced in certain habitats or depth ranges by the challenges of supporting the metabolic demands of a larger body mass under resource-limited conditions such as those typically found in the bathypelagic [26,27].

In addition to body mass, red blood cell counts (RBCs) and the concentration of myoglobin ([Mb]) in muscles represent dimensions over which tissue oxygen reservoirs, and hence dive durations, can vary [19,28,29]. Noren & Williams (2000) [19] showed a positive, although comparatively weak, correlation of maximum dive duration with muscle myoglobin concentrations across a range of taxa within Odontoceti. Mirceta *et al*. (2013) [21] found a similar relationship overall within a broader suite of diving and non-diving mammals. However, there also exist upper limits and trade-offs associated with boosting body oxygen reservoirs by increasing RBCs and/or increasing [Mb]. On the one hand, greater RBCs increase blood viscosity and the cardiovascular pressure necessary to circulate blood [30], and on the other hand myoglobin units may self-adhere and denature as they become more closely spaced within muscle fibers [21,31].

Finally, dive duration may also be extended by tolerating imbalances between total oxygen reservoirs and oxygen consumption during dives, through a transition from aerobic to anaerobic glycolysis pathways in muscle tissues as muscular oxygen reserves become depleted [7,8,32,33]. Extending dive time beyond aerobic dive limits (ADL) faces an ultimate ceiling set by the aerobic metabolic demands of critical organ systems and avoidance of tissue damage from lactic acid accumulation [34]. However, within these overall boundaries, exceeding ADL represents an additional potential evolutionary or facultative behavioral strategy for increasing the range of accessible habitats and/or reducing the ratio of commuting time to foraging time within a dive [7,8,33]. In one of the few species in which ADL has been empirically measured, Weddell seals *Leptonychotes weddellii* completed >92% of dives within ≤26 min, necessitating minimal recovery time (<10 min) between dives during which time gases were exchanged [32]. In contrast, infrequent and opportunistic extended dives ranged up to a maximum of 61.4 min. Extended dives (>26 min) coincided with near-exponential increases in arterial blood lactate concentrations and the extension of recovery periods from ~10 min to ~120 min [32]. This change in the duration of recovery periods highlights a “time efficiency” trade-off for extending dives beyond ADL, in that the proportion of the total time budget available for foraging within deep prey layers is substantially reduced [7,9].

In this study, we collected and analyzed an extensive multi-species satellite telemetry and biologging dataset from the northern Bahamas archipelago. We first quantitatively characterized where different species and individuals in a sympatric assemblage of deep-diving toothed whales foraged in the complex underwater canyons of the Bahamas. We subsequently examined the potential roles of morphological (*e*.*g*., body mass), physiological (*e*.*g*., myoglobin concentration, [Mb]), and behavioral (*e*.*g*., recovery period duration) traits in dive duration capacities and vertical habitat use patterns. We also investigated temporal variation in vertical habitat use over diel cycles, and spatial distribution patterns with respect to bathymetry. Finally, we placed inter-specific differences in habitat use within the context of diving efficiency trade-offs, ecological variation in the vertical and spatial dimensions of prey fields, and relative vulnerabilities to anthropogenic stressors.

## Materials and methods

### Ethics statement

The research presented in this study including tagging and remote biopsy sampling of animals was conducted under a Bahamas Marine Mammal Research Permit (permit #12A) issued to the Bahamas Marine Mammal Research Organisation (BMMRO) by the Government of the Bahamas under authorization of the Bahamas Marine Mammal Protection Act (2005). The specific protocols used in this study, including tag types, methods of deployment, and sample sizes, were approved prior to the start of the study by the US Department of the Navy Bureau of Medicine and Surgery (BUMED) Veterinary Affairs Office and BMMRO’s Institutional Animal Care and Use Committee (IACUC). Annual reviews of the protocols were conducted by BMMRO’s IACUC until the study ended.

### Field data collection

Between 2009 and 2014, tagging and biopsy sampling of deep-diving odontocete cetaceans was conducted from a small boat (6.8 m rigid hull inflatable) in the Great Bahama Canyon system between 23°N and 27°N, and 76°W and 79°W. Cetaceans were located by using visual search from ship and small boat platforms that followed line transect and *ad hoc* survey protocols, and by passive acoustic monitoring with towed and fixed hydrophone arrays [35,36].

Two models of small (49–55 g) satellite telemetry and dive behavior recording tags in the Low Impact Minimally Percutaneous External Electronics Transmitter (LIMPET) configuration were successfully attached to five odontocete species: melon-headed whales *Peponocephala electra*, (Family Delphinidae), short-finned pilot whales *Globicephala macrorhynchus* (Family Delphinidae), sperm whales *Physeter macrocephalus* (Family Physeteridae), Cuvier’s beaked whales *Ziphius cavirostris* (Family Ziphiidae), and Blainville’s beaked whales *Mesoplodon densirostris* (Family Ziphiidae). Location and temperature recording SPOT tags (AM-S240A-C, Wildlife Computers Inc., Redmond, Washington, USA) [37] and location- and dive-depth recording SPLASH tags (Mk-10, Wildlife Computers Inc., Redmond, Washington, USA) [38] were affixed to free-ranging cetaceans using 4–6.5 cm surgical grade titanium darts propelled into the connective tissue on or near the base of cetacean dorsal fins by crossbow [37] or black powder gun [39] from a range of 5–25 m.

### Morphometrics, physiological measurements and demographics

Body mass (*m*) was not measured directly in this study due to the logistical challenge of obtaining this measurement in the free-ranging cetaceans. Body masses of our study species were also not reported in a consistent format across the literature (*e*.*g*., maximum, mean, approximation). To standardize estimates of median body mass for the subsequent modeling of dive durations and dive depths, we drew on standard length and body mass measurements from a variety of stranding and historic whaling records (see S1 Appendix). To estimate median body mass for each sex and age class we first developed models of the relationship between body mass and standard length, and subsequently predicted median body mass from modelled relationship for each species using a comparatively unbiased median standard length estimate (see details in S1 Appendix). Myoglobin concentration ([*Mb*]) was an additional covariate considered in the subsequent modelling of dive durations and dive depths. Species-level mean myoglobin concentrations from epaxial muscles (*longissimus dorsi*) were obtained from a variety of published sources (see details in S1 Appendix).

Tagged individuals were assigned to different sexes and age classes on the basis of sexually dimorphic characteristics, where present (*i*.*e*., *G*. *macrorhynchus*, *Z*. *cavirostris*, *M*. *densirostris*), as well as skin biopsy samples collected using a remote dart biopsy technique [40]. Genetic sex was determined for biopsied whales based on either PCR amplification [41] or real-time PCR [42] of regions of the SRY and ZFX genes. Otherwise, sexes were treated as unknown.

### Spatial distributions and location-specific covariates

During surfacing intervals, both SPOT and SPLASH tags transmitted a series of messages to overhead Argos satellites (http://www.Argos-system.org). The movement track for each tagged cetacean over a tag’s transmission life was estimated from irregularly spaced Argos position fixes using a Continuous Time Correlated Random Walk (CTCRW) [43] model fitted in the R package *crawl* [44], with subsequent modifications to allow the explicit inclusion of Argos error ellipse extent and shape [45]. Location estimates were predicted from fitted CTCRW models at regular one-hour date-time stamps within each track, and were subsequently used to: 1) calculate solar and lunar rise and set times 2) estimate bathymetric depth and 3) predict the isotherm boundaries at the mean locations of SPOT time-at-temperature histograms (see *Dive Patterns*). Bathymetric depths were extracted from a 0.0083° latitude and longitude resolution bathymetric digital elevation model at predicted locations using the function *extract* from the R library *raster* [46]. Sunrise and sunset times, as well as solar angular elevations, were calculated at the predicted locations and corresponding date-time stamps using the function *sunriset* from the R library *maptools*.

### Dive depth, dive duration, and inter-deep-dive intervals

Messages from tags used for location estimation also included internally summarized dive behavior data. These moderate-resolution summaries of raw high-resolution environmental sensor outputs (*e*.*g*., temperature, pressure, wet/dry) facilitated transmission over bandwidth- and time-limited connections with Argos satellites. In SPLASH tags, a pressure sensor allowed the direct measurement of dive depth and dive duration. Pressure transducer observations (accuracy: ±1% of depth reading) from SPLASH tags were compressed and transmitted in the form of: 1) a behavior log which summarized pressure and wet-dry measurements into sequences of dives and surface intervals, and 2) a time-series log that recorded depth observations at either 2.5 or 5 minute intervals. Each dive in the behavior log was defined by the deepest depth and time interval between successive dry measurements. In contrast, SPOT tags carried a thermistor and transmitted time-at-temperature (TAT) histogram summaries, as a proxy for dive depth activity [47]. TAT summaries consisted of the proportion of thermistor readings, collected at 10-second intervals over 6-hour sampling periods, that fell into 12 temperature categories (<4°C, 4–6°C, 6–8°C, 8–10°C, 10–12°C, 12–14°C, 14–16°C, 16–18°C, 18–20°C, 20–22°C, 22–24°C and ≥24°C). Sampling periods of TAT were programmed to begin at 01:00, 07:00, 13:00, or 21:00 local time, so that the majority (>80%) of sampling of each TAT histogram fell within either daytime or nighttime periods.

“Foraging” dives were defined as dives falling within species-specific depth ranges where foraging activity was known or inferred to occur. Dive distributions were principally interpreted using published digital acoustic recording tag (DTAG) dive profiles that described the vertical distribution of echolocation clicks and/or “buzz” vocalizations associated with prey capture attempts [3–5,33,48]. Published DTAG profiles were available for all tagged study species except *P*. *electra*. In particular short and relatively shallow dives among beaked whales, labeled “bounce dives” by Tyack *et al*. (2006) [33], have been shown to be non-foraging in nature based on the absence of acoustic foraging cues. The time intervals between successive “foraging” dives in the behavior log were referred to as inter-deep-dive intervals (IDDI) following the definition used by Tyack *et al*. (2006) [33] and synonymous with the inter-foraging dive interval (IFDI) defined by Arranz et al. (2011) [5]. These were calculated for all species as the sum of surface period durations interspersed between deeper dives plus the duration of any short and comparatively shallow dives that did not meet the foraging dive criteria. We further defined “time efficiency” as the proportion of each tagged individual’s time budget spent within foraging depth strata.

### Models of dive duration and depth

Several previous inter-specific comparative analyses of mammalian dive behaviors [19–21] have examined factors influencing maximum dive duration (*T*_*max*_) as a response variable. As an initial step in examining the morphological, physiological, and behavioral factors influencing dive behavior variation in the odontocete assemblage of our study area, we evaluated the predictive performance of the odontocete-specific allometric model (Eq 2) from Noren & Williams (2000) [19].

$$T_{max}=0.68\;m^{0.47}$$ (1)

For comparison with previous modeling efforts, we developed models of *T*_*max*_ as a function of body mass (*m*) using dive information from the tagged individuals in this study. In using individual level data, we had to account for intraspecific non-independence of dive behaviors and for phylogenetic interdependencies among species. Random effects structures in a mixed-model framework can be used to account for non-independence in model residuals [49]. Villemereuil and Nakagawa (2014) [49] further elaborated an approach to explicitly account for the evolutionary interdependencies among species; they integrate a phylogenetic tree model within the mixed-model framework called phylogenetic generalized linear mixed models (PGLMM). We developed PGLMM of *T*_*max*_ incorporating the cetacean phylogenetic tree and divergence time estimates of McGowen, Spaulding & Gatesy (2009) [50], as well as three fixed-effects covariates: estimates of median body mass (*m*, see S1 Appendix), literature-derived mean myoglobin concentrations ([*Mb*], see S1 Appendix), and median IDDI calculated from the behavior log dataset. Because models of *T*_*max*_ potentially unrealistically collapsed all of the variation within each individual’s dive behavior to a single number, we also developed log-log PGLMM models of dive duration (*T*) and dive depth (*Z*) at the level of individual dives in S2 Appendix.

Following the mixed-effects model selection guidance outlined by Zuur *et al*. (2009) [51], we first contrasted random-effects structures and a fixed-effects-only model each containing all three fixed-effects covariates. Bayesian posterior probability distributions for a fixed-effects-only log-log (*i*.*e*., power law) generalized linear model (GLM) and a log-log PGLMM model of *T*_*max*_, were implemented in the R function *MCMCglmm* [49]. Bayesian Markov Chain Monte Carlo (MCMC) simulations used the default Gaussian fixed-effects prior (mean = 0 and variance = 10^10^) implemented in *MCMCglmm*, and an inverse-Gamma random-effects prior with shape and scale parameters of 0.01 [49]. MCMC simulations were repeated over 10^5^ iterations, discarding a burn-in phase of 1000 iterations and applying a further thinning of 1 in every 50 iterations. In this initial phase of comparing model random-effects structures, the fixed-effects and mixed-effects log-log models were evaluated on the basis of Deviance Information Criterion (DIC) scores, and a model weighting metric, *w*DIC, that was developed based on the Akaike’s weight metric (*w*AIC) from Burnham & Anderson (2002) [52].

$$wDIC=\frac{e^{-0.5({DIC}_{i}-{DIC}_{min})}}{\sum_{i=1}^{n}e^{-0.5({DIC}_{i}-{DIC}_{min})}}$$ (2)

After the selection of a random-effects structure (in this case the fixed-effects-only model), log-log PGLMM with the different combinations of fixed-effects terms were fitted, and a second phase of model selection was undertaken on the basis of DIC and *w*DIC.

To provide a broader phylogenetic context for the dive behaviors observed among the five tagged species in this study area, we also developed phylogenetic generalized least squares (PGLS) [53] models of *T*_*max*_. This analysis incorporated a range of other odontocete species for which we found literature-reported values of *T*_*max*_, *m*, [*Mb*]_,_ and IDDI or were able to extract these values from supplemental materials. The additional species included: belugas *Delphinapterus leucas* [19,54], narwhals *Monodon monoceros* [55], harbour porpoise *Phocoena phocoena* [56], killer whales *Orcinus orca* [57], pantropical spotted dolphins *Stenella attenuata* [58], pygmy sperm whales *Kogia breviceps* [59,60], and northern bottlenose whales *Hyperoodon ampullatus* [61]. Like PGLMM, PGLS models in this analysis incorporated the McGowen, Spaulding & Gatesy (2009) [50] phylogenetic tree into the variance-covariance matrix of a GLS model using a Brownian motion model of trait evolution [53].

### Foraging depth and bathymetric depth

Telemetry data from all tagged individuals was used to examine the spatial overlap of different species and sexes with habitats where the benthos was accessible within the observed foraging dive range of each species. Because the number of position fixes recovered for each individual varied, species averages of the proportion of coordinate fixes falling within accessible benthic habitats were calculated as a grand mean of averages for each individual. Finally, we calculated Pearson correlation coefficients between the maximum depth of dives recorded in the behavior log for each species and the bathymetric depth corresponding to the CTCRW predicted location at the midpoint of each dive.

## Results

### Tagging

Between 2009 and 2014, 75 Argos tags were successfully deployed on five deep-diving odontocete cetacean species commonly encountered within our study area, yielding 7752 position fixes and 12,204 h of dive data (Table 1). Both SPOT and SPLASH tags were successfully deployed on all five species (Table 1), but SPLASH tags were preferentially used on beaked whales resulting in higher proportions of SPLASH tag deployments (*M*. *densirostris* 75% and *Z*. *cavirostris* 86%). Tissue biopsies were obtained from 24 individuals during the tagging encounter. Cross-referencing tagged individuals with photo-identification catalogs identified an additional 15 tagged whales that were biopsied and tagged on separate occasions. The overlap between tagging and biopsy datasets provided genetic sex for 52% of all tagged individuals, with *P*. *electra* representing the majority of individuals for which sex was unknown.

**Table 1:** Number of satellite tag deployments between 2009–2014, by tag type and sex. Tagging events indicate the number of separate encounters with groups of odontocetes during which tagging took place; some groups may have been repeatedly encountered across multiple years. Also shown are the total hours of dive data recovered for each species in the behavior and time series logs of SPLASH tags, time-at-temperature histograms of SPOT tags, and mean duration of tag transmission for five species of deep-diving odontocetes in the northern Bahamas.

Species	SPOT M F U	SPLASH M F U	Tagging Events	Behav. Log (h)	Time Series (h)	TAT Histo. (h)	Mean Duration d (Min, Max)
Melon-headed whale *Peponocephala electra*	0 0 9	0 0 4	6	55.45	15.04	1605	9.51 (0.02, 38.79)
Short-finned pilot whale *Globicephala macrorhynchus*	7 4 1	2 0 1	5	252.61	277.83	3639	15.67 (0.37, 40.8)
Sperm whale *Physeter macrocephalus*	11 9 1	5 1 0	13	451.01	140.42	3340	8.2 (0, 17.76)
Blainville’s beaked whale *Mesoplodon densirostris*	3 0 0	3 6 0	10	695.91	694.75	167	16.41 (0.01, 45.64)
Cuvier’s beaked whales *Ziphius cavirostris*	0 1 0	4 2 0	6	365.65	139.96	139	23.72 (8.53, 90.14)

SPOT, temperature-only (AM-S240A-C) satellite tag; SPLASH, depth-and-temperature (Mk-10) satellite tag; M, male; F, female; U, unknown sex; h, hours; d, days

### Dive depth and duration

**Fig 1:**
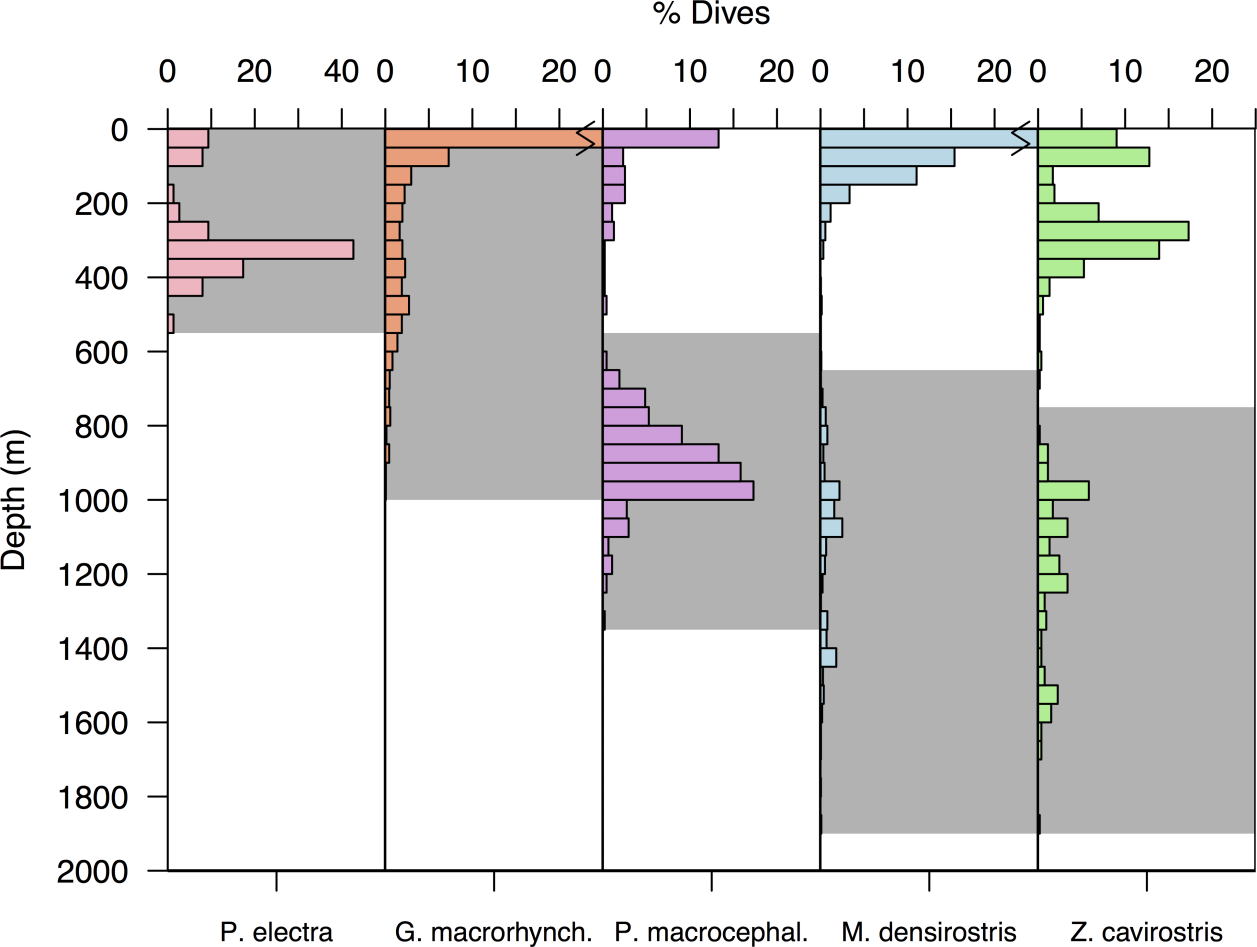
Histograms showing the vertical distribution of foraging dive depths in five deep-diving odontocete species. The dives that were classified as probable foraging dives on the basis of the vertical distribution of foraging buzzes recorded in prior digital acoustic recording tag studies [3–5,33], which are indicated by the grey background colours.

**Fig 2:**
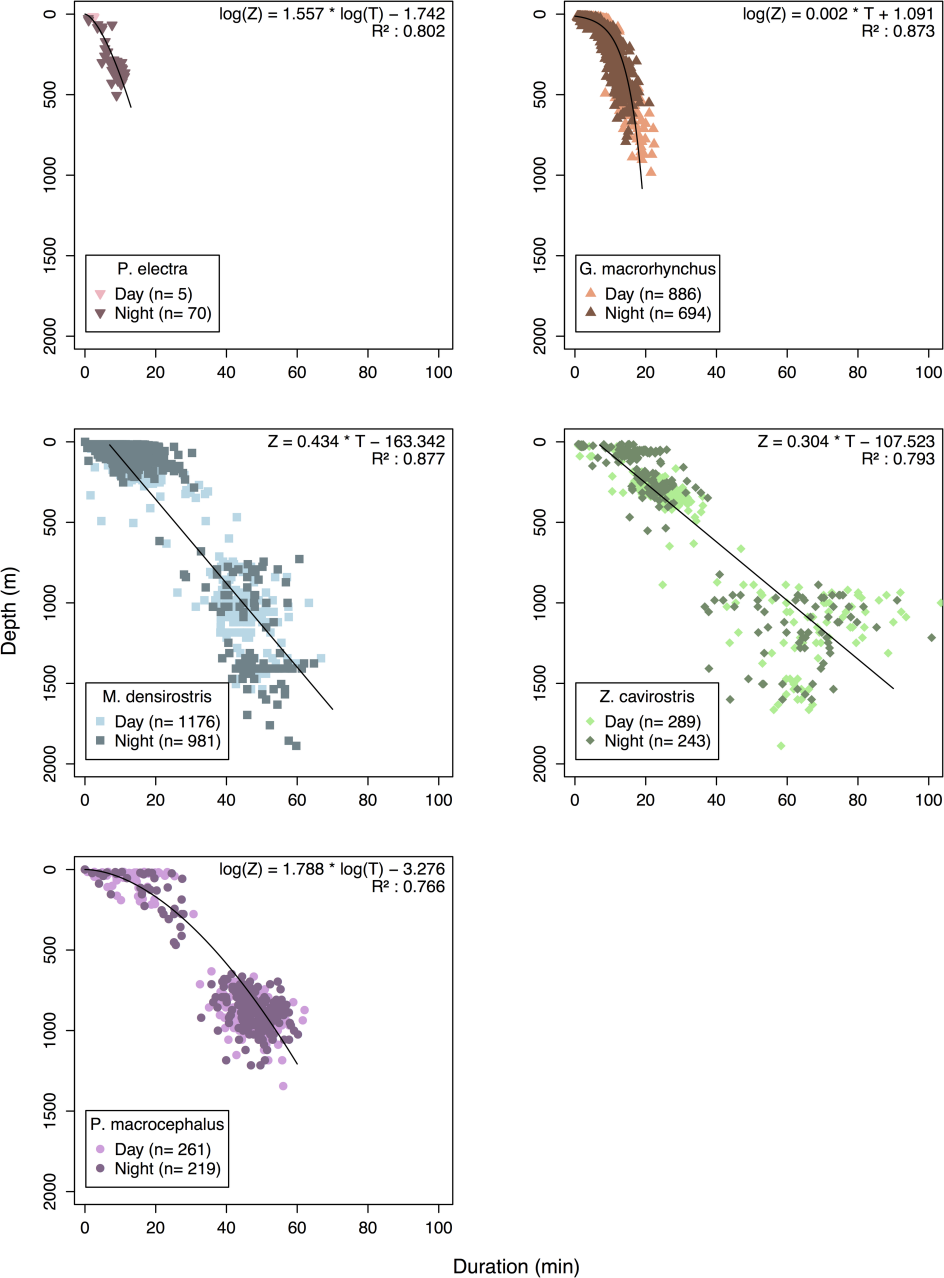
Scatter plots showing the relationship of dive duration to dive depth in five deep-diving odontocete species. Dive duration generally increased as a function of dive depth in this study. However, different functional forms ranging from exponential (*Globicephala macrorhynchus*), to power law (*Peponocephala electra* and *Physeter macrocephalus*), to linear (*Mesoplodon densirostris* and *Ziphius cavirostris*) provided the best model fits in different species. Greater variation in dive duration of deeper dives was observed particularly among the beaked whales.

The tagged species occupied partially overlapping foraging ranges (Fig 1) in the vertical dimension, ranging from the epipelagic (<200m) and upper mesopelagic (~200–800 m) and among *P*. *electra* and *G*. *macrorhynchus* to the lower mesopelagic and upper bathypelagic in *P*. *macrocephalus*, *M*. *densirostris* and *Z*. *cavirostris*. Overall, dive depths were positively related to dive duration in each species, although the relationship was non-linear in three of five tagged species. Power-law and exponential functional forms provided the best fits for *P*. *electra*, *G*. *macrorhynchus*, and *P*. *macrocephalus*, while linear formulations provided better fits for *M*. *densirostris* and *Z*. *cavirostris* (Fig 2).

### Models of maximum dive duration

**Fig 3:**
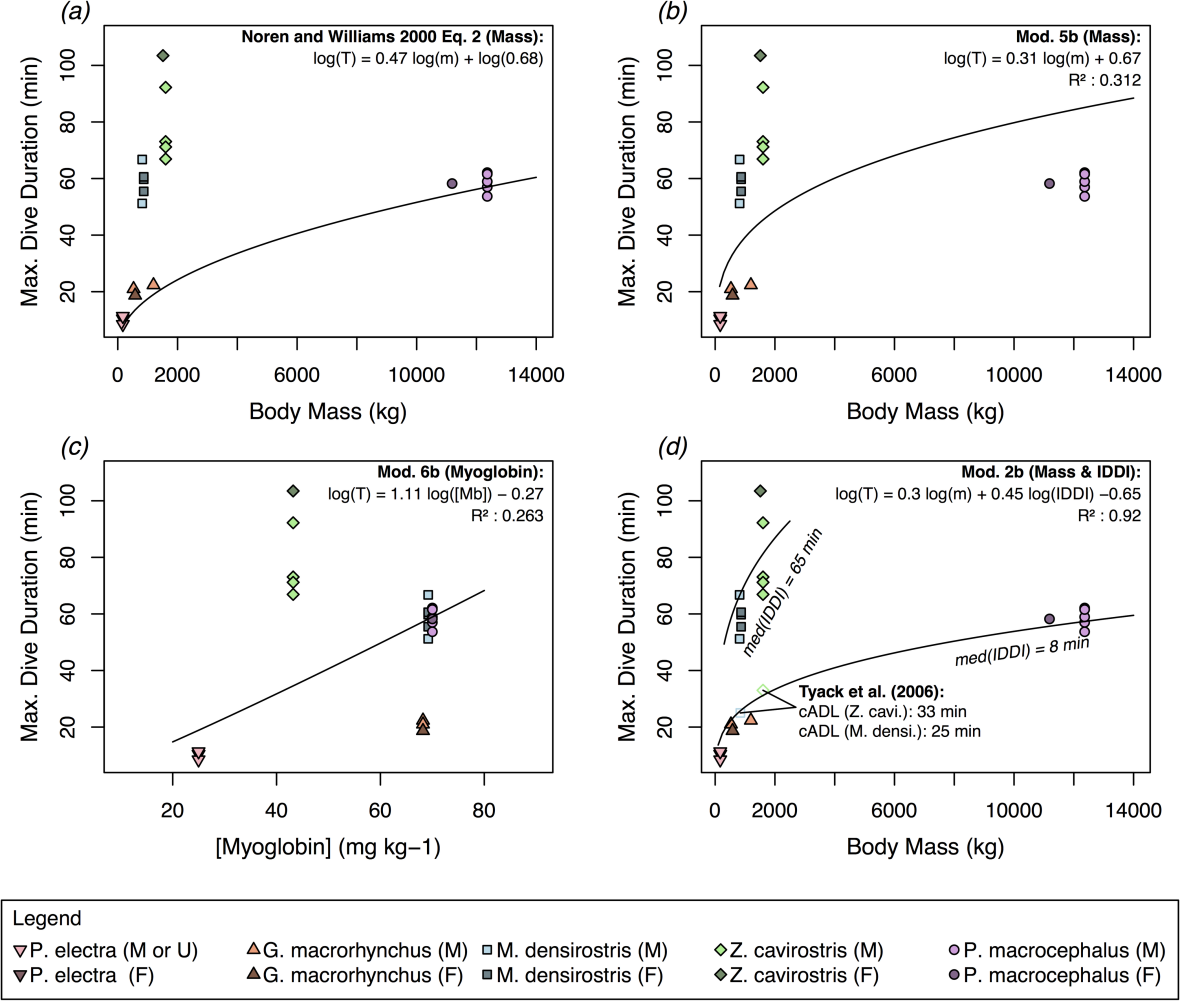
Relationship of maximum dive duration (*T*_*max*_) to body mass (*m*) and myoglobin concentration ([*Mb*]). *T*_*max*_ in this plot were based on the longest dive recorded by each individual tagged with a SPLASH model satellite transmitter. The solid line in sub-plot (*a*) shows the power law relationship (Eq 2) fitted by Noren and Williams (2000) [19]. This trendline provides relatively close fit to *T*_*max*_ among the delphinids and physeterids, but substantially underestimates *T*_*max*_ of the two ziphiids. The two trendlines shown in sub-plot (*d*) indicate the different predicted relationships between *T*_*max*_ and *m* based on different median inter-deep dive interval (IDDI) values among the delphinids and physeterids of 8 min and among the ziphiids of 65 min. Also shown on sub-plot (*d*) are the calculated Aerobic Dive Limit (cADL) values estimated by Tyack et al. (2006) [33] for *Mesoplodon densirostris* and *Ziphius cavirostris*.

In examining the morphological, physiological, and behavioral factors influencing maximum dive duration in our study species, we found that models employing *m* and [*Mb*] alone provided inadequate explanations of the observed variation in *T*_*max*_ among the tagged individuals in this study. The variance of the observed *T*_*max*_ values in this study minus the *T*_*max*_ values predicted by Eq 2 in Noren & Williams (2000) [19] was larger than the variance associated with the mean of the observed values ($\bar{T_{max}}$). This equation therefore explained less variance than an overall mean (i.e., R^2^ = 0). In particular, this model substantially underestimated maximum dive duration in the two ziphiid species *M*. *densirostris* and *Z*. *cavirostris* (Fig 3A). However, it did perform considerably better when predicting maximum dive duration of individuals in the delphinid (*P*. *electra* and *G*. *macrorhynchus*) and physeterid (*P*. *macrocephalus*) families (R^2^ = 0.90, Fig 3A). This divergence from expectations of simple allometric scaling of *T*_*max*_ was highlighted by the maximum dive duration of *M*. *densirostris*, which on average exceeded the maximum dive durations of male *G*. *macrorhynchus* by a factor of 5.5x, despite having a 30% smaller median mass (*M*. *densirostris*: 842.9 kg, *G*. *macrorhynchus*: 1195.4 kg). Additionally, despite weighing 7.6x less than *P*. *macrocephalus*, *Z*. *cavirostris* dove an average of 17.6 min longer per deep dive. Because of these large disparities, a simple allometric GLM model (Mod. 5b, Table 2) did not fare substantially better than the Noren & Williams allometric model in predicting *T*_*max*_ of tagged individuals, which can be seen in a relatively poor trend line fit (Fig 3B), and in an R_m_^2^ value of 0.31 (Table 2). The log-log GLM model with [*Mb*] as covariate (Mod. 6b, Table 2) likewise explained only 26% of the variance in *T*_*max*_, which is similarly indicated by a weak trend line fit (Fig 3C). Together *m* and [*Mb*] accounted for <37% of the variance in *T*_*max*_.

**Table 2:** Comparison of models describing variation in the maximum dive duration. The first section (above solid line) compares a fixed-effect only model with a mixed model, which includes random effects of species with a phylogenetic correlation structure. The second section (below solid line) compares different combinations of the fixed effect covariates within the model structure selected in the first section. Marginal and conditional coefficients of determination indicate the proportion of variance explained by the fixed effects only and the full mixed effects model, respectively [62].

Model Formula	*k*	*R*_*m*_^2^	*R*_*c*_^2^	DIC	ΔDIC	*w*DIC
Mod. 1a (GLM): log(*T*_max_) ~ log(*m*) + log([*Mb*]) + log(*IDDI*)	4	0.91	-	-41.91	0	0.71
Mod. 2a (PGLMM): log(*T*_max_) ~ log(*m*) + log([*Mb*]) + log(*IDDI*) | *SPP*	5	0.36	0.95	-40.15	1.76	0.29
Mod. 1b (GLM): log(*T*_max_) ~ log(*m*) + log([*Mb*]) + log(*IDDI*)	4	0.92	-	-41.84	2.3	0.24
Mod. 2b (GLM): log(*T*_max_) ~ log(*m*) + log(*IDDI*)	3	0.92	-	-44.14	0	0.76
Mod. 3b (GLM): log(*T*_max_) ~ log(*m*) + log([*Mb*])	3	0.36	-	8.54	52.68	0
Mod. 4b (GLM): log(*T*_max_) ~ log(*IDDI*) + log([*Mb*])	3	0.69	-	-10.21	33.93	0
Mod. 5b (GLM): log(*T*_max_) ~ log(*m*)	2	0.31	-	8.46	52.6	0
Mod. 6b (GLM): log(*T*_max_) ~ log([*Mb*])	2	0.26	-	10.38	54.52	0
Mod. 7b (GLM): log(*T*_max_) ~ log(*IDDI*)	2	0.58	-	-3.85	40.29	0

GLM, generalized linear models; PGLMM; phylogenetic generalized linear mixed models; *T*_max_, maximum dive duration; *SPP*, species; *m*, body mass; [*Mb*], myoglobin concentration; *IDDI*, inter-deep-dive interval; DIC, Deviance Information Criterion; ΔDIC, DIC difference; *w*DIC, DIC weights [52]; *k*, number of model parameters; *R*_*m*_^2^, marginal coefficient of determination [62]; *R*_*c*_^2^, conditional coefficient of determination [62].

**Fig 4:**
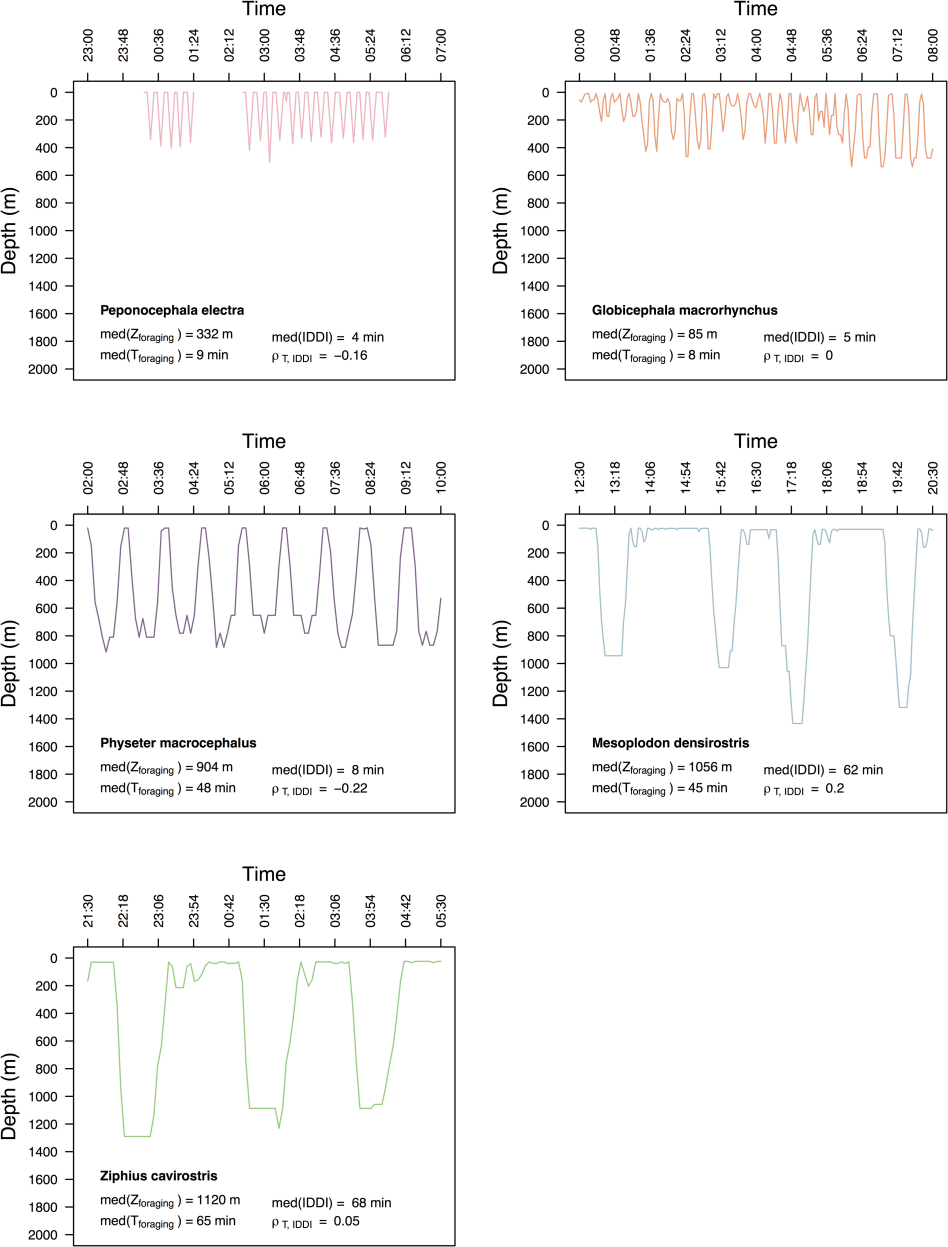
Typical time-depth dive profiles from SPLASH tags deployed on five deep-diving odontocete species. Each plot shows a moderate-resolution satellite-transmitted record representing eight hours of dive behavior (note: *Peponocephala electra* record shows several gaps in transmission). This panel contrasts the comparatively short inter-deep dive intervals (IDDI) and relatively continuous diving of the delphinids and physeterids, with the longer dive durations and longer IDDI of the ziphiids. The median depth (*Z*_*foraging*_), median duration (*T*_*foraging*_), median surface intervals (*IDDI*), and correlation coefficients of duration and IDDI (ρ _*T*, *IDDI*_) are shown across the bottom of each plot.

**Fig 5:**
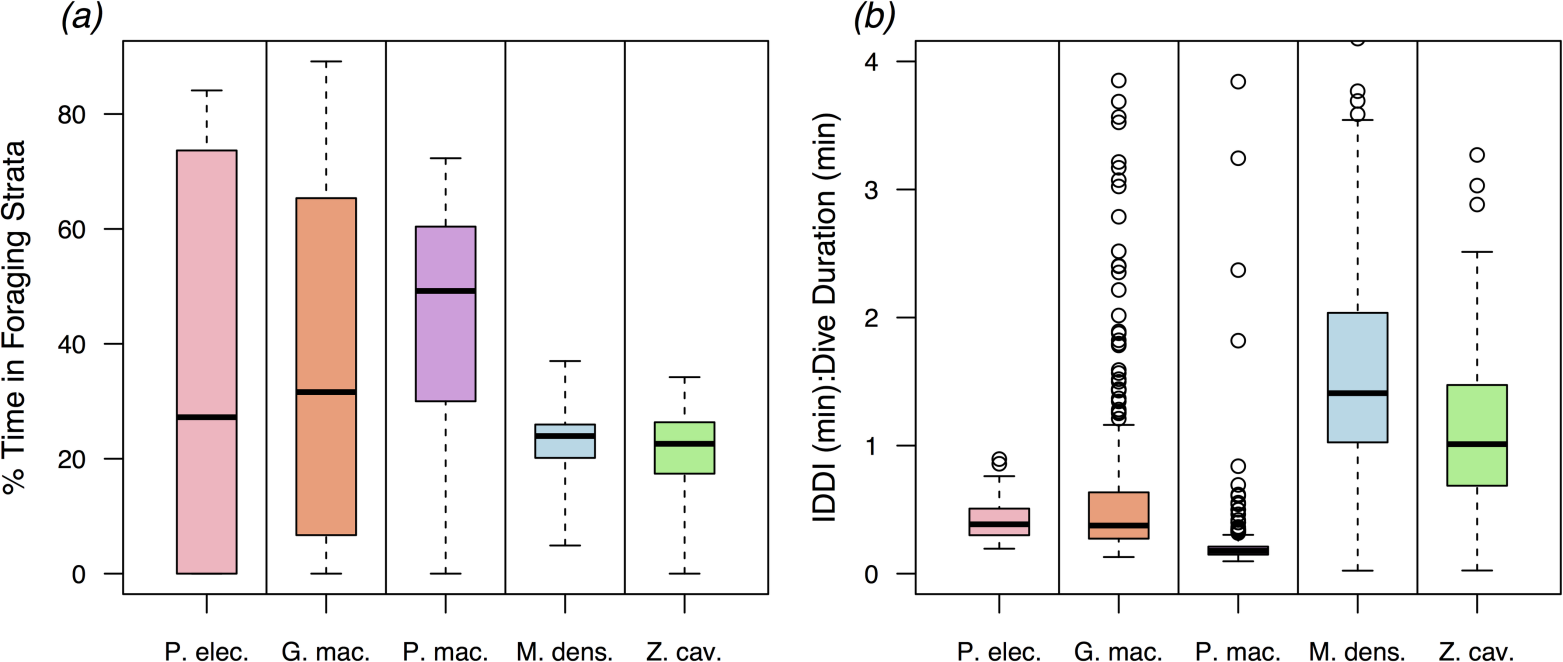
**Boxplots illustrating (*a*) the proportion of time within foraging depths and (*b*) and the ratio of inter-deep dive intervals (IDDI) to dive duration (*T*).** The proportions of time spent in foraging strata derived from SPOT tag time-at-temperature histograms that were translated into units of depth using the hydrographic data and interpolation methods detailed in Joyce *et al*. (2016) [47]. Overall, the ziphiids exhibited a substantially lower proportion of time spent in foraging strata relative to delphinids and physeterids. This difference resulted primarily from the substantially longer inter-deep dive intervals (IDDI) among by the ziphiids, which is shown by the ratios of IDDI to dive duration (*T*) in plot (*b*). Among the ziphiids IDDI were typically greater than or equal to *T*, while among delphinids and physeterids IDDI were typically substantially less than *T*.

Including differences in IDDI shown in Fig 4, along with *m* in log-log GLM models (Mod 2b, Table 2) explained a substantially greater proportion of the variance in *T*_*max*_ (R_m_^2^ = 0.92) relative to models with *m* and/or [*Mb*]. This improvement in model fit was demonstrated by: 1) the selection of Model 2b (IDDI and *m*) as the most parsimonious model fit (Table 2 and 2) the trend lines in Fig 3D, which show the different functional relationships of *T*_*max*_ to *m*, with input values of IDDI corresponding to the median IDDI of the delphinids and physeterids (8 min), and ziphiids (65 min). In addition to the IDDI of the two ziphiids being comparatively long in absolute terms (Fig 4), these IDDI were also long in proportion to dive duration (Fig 5B), with median IDDI: *T*_*max*_ ratios of 1.41 and 1.01 among the ziphiids (*M*. *densirostris* and *Z*. *cavirostris*, respectively) compared to ratios of 0.39, 0.38, and 0.17 among the delphinids and physeterids (*P*. *electra*, *G*. *macrorhynchus*, and *P*. *macrocephalus*, respectively).

**Fig 6:**
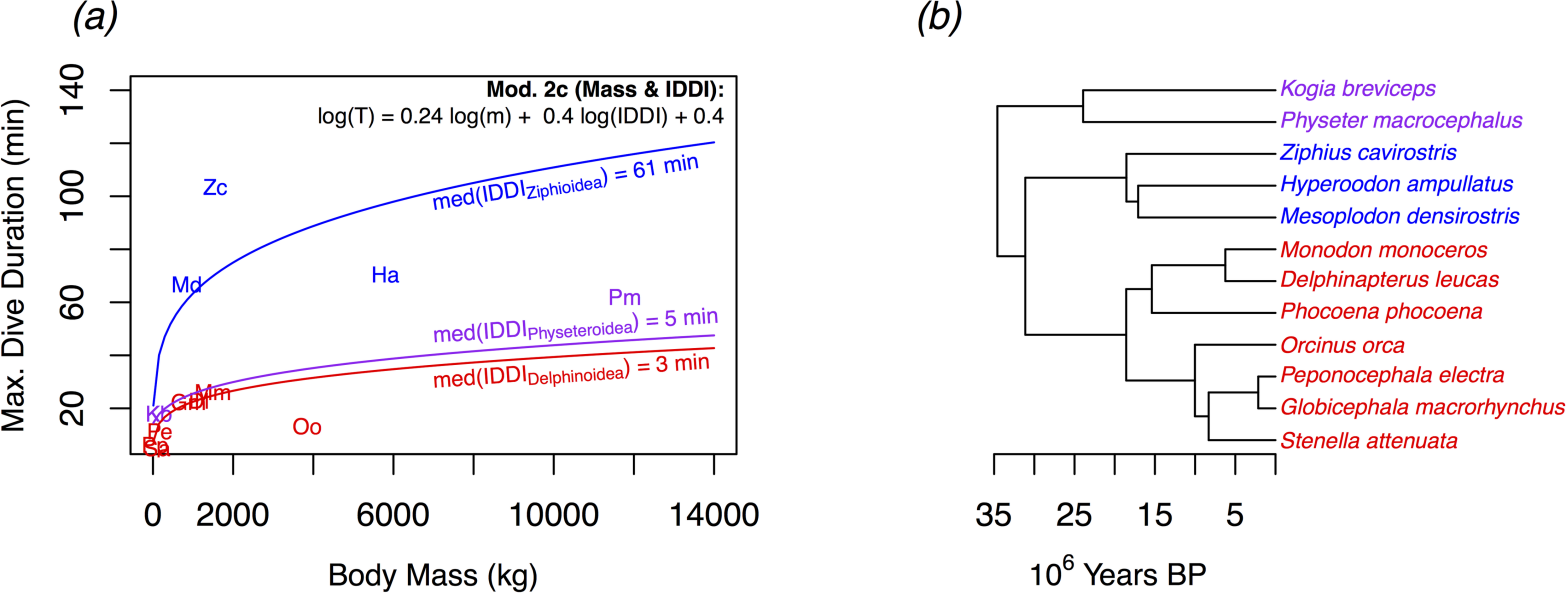
Phylogenetic generalized least squares (PGLS) model of maximum dive duration (*T*_*max*_) in 12 odontocete species. Covariates of *T*_*max*_ includes body mass (*m*), myoglobin concentration ([*Mb*]), and inter-deep-dive interval (IDDI). The three curves overlaid on this scatterplot (*a*) represent the predictions from the PGLS Model 2c (Table 3) using the median IDDI values of the super-families: *Ziphioidea* (blue), *Physeteroidea* (purple), and *Delphinoidea* (red). These super-families are also indicated in the phylogenetic tree hypothesis of McGowen, Spaulding & Gatesy (2009) [50] (*b*), which was used to develop the correlation structure in PGLS models. Species in (*a*) are indicated by the first letters of the genus and species name (e.g., *Physeter macrocephalus* = Pm).

The comparison of PGLS models in Table 3 further supported the importance of IDDI in the prediction of *T*_*max*_, in this case over a wider range of taxonomic diversity than the species tagged in the present study. This analysis included representative species from all six primarily marine (*i*.*e*., excluding river dolphins) families of Odontoceti. Fig 6 also shows a clustering of species belonging to the Delphinoidea (Families: Monodontidae, Phocoenidae, Delphinidae) and Physeteroidea (Families: Kogiidae, and Physeteridae) superfamilies in *m* and *T*_*max*_ space, while the Ziphioidea (Family: Ziphiidae) were clearly clustered apart from these two superfamilies.

**Table 3:** Comparison of phylogenetic generalized least squares (PGLS) models describing variation in the maximum dive duration (*T*_max_) across 12 odontocete species. The top three candidate PGLS models all included IDDI (Models 1c, 2c, & 4c), and together accounted for 92% of wAIC probabilities.

Model Formula	*k*	AIC	ΔAIC	wAIC
Mod. 1c (PGLS): log(*T*_max_) ~ log(*m*) + log([*Mb*]) + log(*IDDI*)	4	-2.06	0	0.52
Mod. 2c (PGLS): log(*T*_max_) ~ log(*m*) + log(*IDDI*)	3	-1.06	1	0.31
Mod. 3c (PGLS): log(*T*_max_) ~ log(*m*) + log([*Mb*])	3	3.65	5.71	0.03
Mod. 4c (PGLS): log(*T*_max_) ~ log(*IDDI*) + log([*Mb*])	3	1.39	3.45	0.09
Mod. 5c (PGLS): log(*T*_max_) ~ log(*m*)	2	3.32	5.38	0.04
Mod. 6c (PGLS): log(*T*_max_) ~ log([*Mb*])	2	9.93	11.99	0
Mod. 7c (PGLS): log(*T*_max_) ~ log(*IDDI*)	2	5.64	7.7	0.01

PGLS, phylogenetic generalized least squares models; *T*_max_, maximum dive duration; *SPP*, species; *m*, body mass; [*Mb*], myoglobin concentration; *IDDI*, inter-deep-dive interval; AIC, Akaike’s Information Criterion; ΔAIC, AIC difference; *w*AIC, Akaike’s weights [52]; *k*, number of model parameters.

### Time budgets

Relative to the ziphiids, the delphinids and physeterids spent a comparatively low proportion of their time budgets at or near the surface during foraging periods and consequently a higher proportion of their time at foraging depths. This differences is illustrated by the maxima of only 37.0% and 34.2% of 6-hour TAT histogram time blocks that were spent by *M*. *densirostris* and *Z*. *cavirostris* individuals in temperature/approximate depth ranges thought to be associated with foraging activity (Fig 5A). This contrasted with upwards of 84.1%, 89.2%, and 72.3% that *P*. *electra*, *G*. *macrorhynchus*, and *P*. *macrocephalus* were able to spend in their respective foraging temperature/approximate depth ranges (Fig 5A). In part, this reflected the shorter commuting distances and consequently shorter commuting times in the shallower diving delphinids and physeterids, relative to the deeper diving ziphiids. However, in bouts of *P*. *macrocephalus* and *M*. *densirostris* dives that reached similar depths (800m), *P*. *macrocephalus* spent an average of 2.05x more of their time engaged in foraging dives relative to *M*. *densirostris*. Averaged over an entire diel cycle, the two delphinid species (*P*. *electra* and *G*. *macrorhynchus*) spent an average of 27.2% and 31.6% of their respective time budgets within target foraging strata, only moderately higher than the 24.0% and 22.6% spent by the two ziphiids (*M*. *densirostris* and *Z*. *cavirostris*, respectively; Fig 5A). However, the low median values and large variability in time efficiency exhibited by these two delphinid species (Fig 5A) predominately reflected the large portions of daylight periods spent at or near the surface when not engaged in foraging behaviors.

### Diurnal patterns

**Fig 7:**
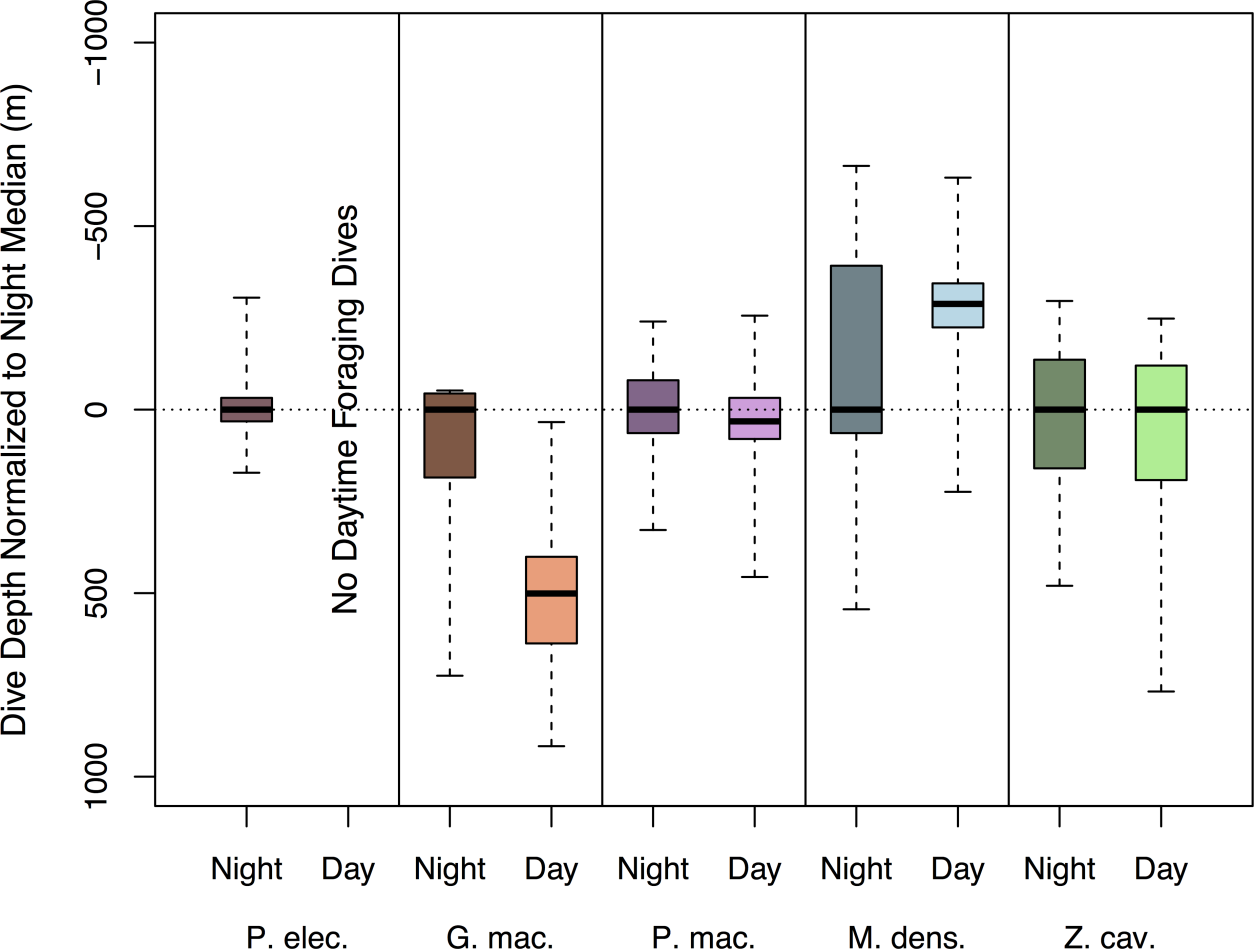
Boxplots showing daytime and night-time foraging dives normalized to median night-time dive depth. Positive values indicate that daytime dives were on average deeper than night-time dives. Although the species-specific patterns of responsiveness to light are complex there is a general trend, from arguably the largest and completely binary response of the shallowest diving species *P*. *electra*, to the deepest diving species, *Z*. *cavirostris*, with no observed response.

The tagged species in this study showed divergent responses to light levels, both in terms of dive depth and dive frequency (Fig 7). The shallowest diving species, *P*. *electra*, showed a binary response, with the absence of daytime dives below 25m or the 24°C isotherm (median depth 117 m) in over 869 hours of daytime behavior log and TAT data. Foraging dive activity in this species appears to be exclusively confined to nighttime periods. *G*. *macrorhynchus*, with the second shallowest median dive depths, undertook daytime dives that were on average, 417 m deeper and 69.9% less frequent than nighttime dives and appear to be concentrated during hours of lower incident light angles (early morning and late afternoon). Unlike any other species tagged in this study, the distribution of nighttime dives in *G*. *macrorhynchus* lacked bi-modality, with a continuous distribution from the central mesopelagic zone to near the surface. However, the nighttime dive pattern of *G*. *macrorhynchus* did differ between the sexes of this highly sexually dimorphic species, with adult males (2.05x larger by mass) showing greater consistency between daytime and nighttime dive depths, in contrast to greater variation between daytime and nighttime foraging dive depths of the smaller-bodied females. Overall *P*. *macrocephalus* displayed small diurnal differences between median daytime (920 m) and nighttime (888 m) dive depths. This pattern also varied among sexes and age classes: females and juvenile males that were tagged within matrilineal social groups dove on average to 3.5% shallower depths and exhibited a 26.4% larger diurnal difference in dive depths when compared with sub-adult males that were tagged either solitarily or in bachelor groups. *M*. *densirostris* exhibited daytime dives that were on average 142.3 m or 11.6% shallower than nighttime dives. This diurnal difference in dive depths was not observed in all tagged *M*. *densirostris*, but was detected in 5 of 7 individuals. *Z*. *cavirostris*, the deepest diving species, showed little to no variability in foraging dive depths between day and night.

### Spatial habitat use

**Fig 8:**
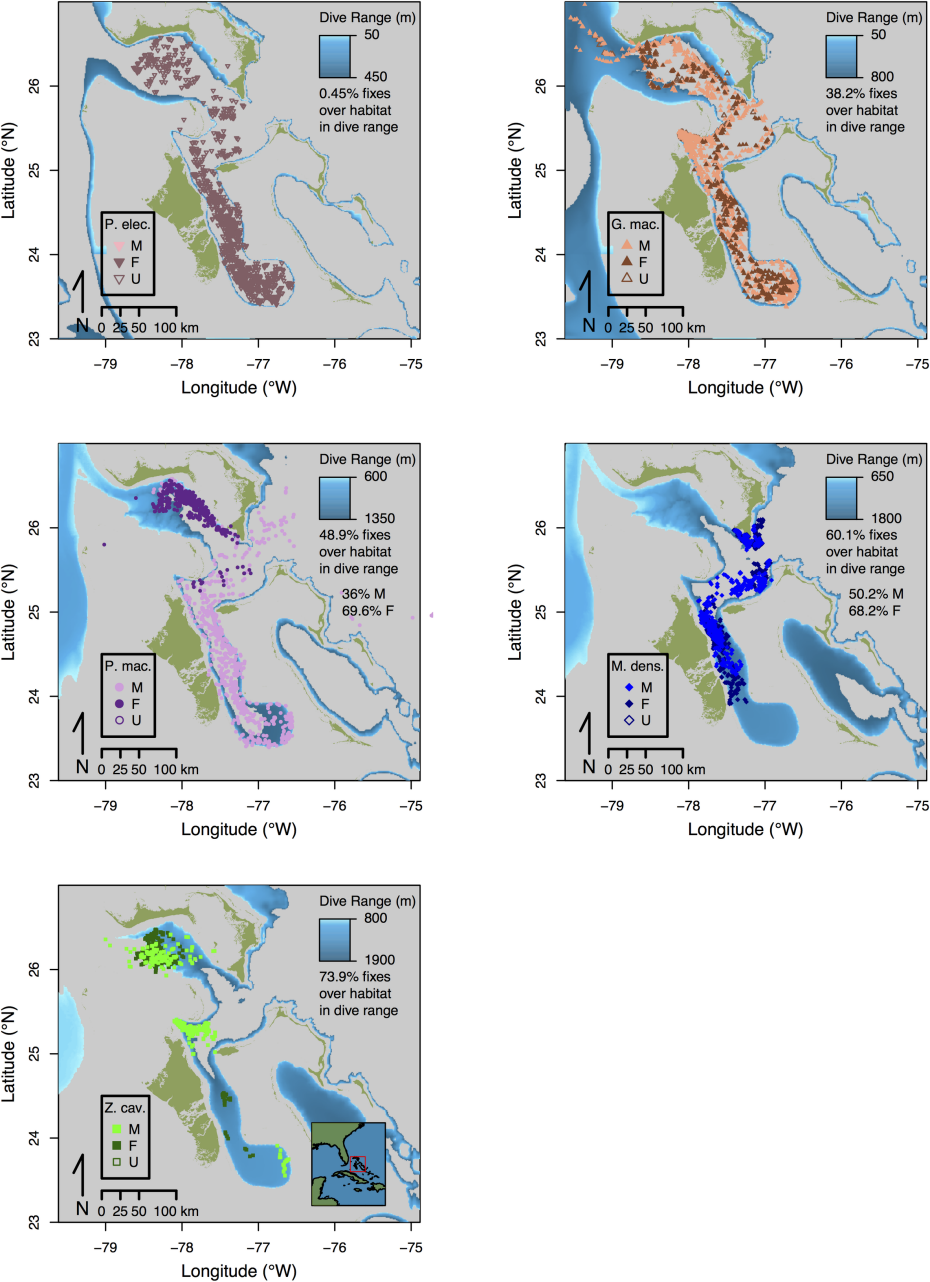
Argos tag locations shown on maps of benthic habitats falling within species-specific foraging dive depth ranges. Locations derive from a continuous time correlated random walk model fitted to Argos locations estimates. The distribution of benthic habitats shown in blue shading correspond to foraging dive depth ranges delineated in Fig 1. Shallower diving species *Peponocephala electra* and *Globicephala macrorhynchus*, as well as sub-adult male *Physeter macrocephalus* show less spatial overlap with accessible benthic habitats. Deeper-diving *Mesoplodon densirostris*, *Ziphius cavirostris* and female *Physeter macrocephalus* show greater spatial overlap, which is quantitatively indicated by percent overlap scores on each map.

Considering the bathymetric topography in our study area, we observed interspecific differences in spatial distribution that were reflected in overlap of CTCRW-predicted position fixes with areas of different bathymetric depth (Fig 8). In general, the CTCRW-predicted locations of the deeper diving species (*P*. *macrocephalus*, *M*. *densirostris*, and *Z*. *cavirostris*) were more frequently localized over areas where the bottom was within reach during their dives (Fig 8C, 8D and 8E), while shallower diving delphinids (*P*. *electra* and *G*. *macrorhynchus*) were proportionally less frequently localized over habitats where the benthos fell within their respective dive depth ranges (Fig 8A and 8B).

There was also some intra-specific variation in these patterns. In particular, *M*. *densirostris* showed considerable inter-individual variation. Nine of 11 *M*. *densirostris* individuals exhibited consistent associations with areas where the bathymetric depth was less than the maximum recorded dive depth of this species (1888m), while the remaining two individuals (1 male and 1 female) ranged widely over a variety of benthic depths that were beyond the range of their dive capacities (Fig 8D). *P*. *macrocephalus* exhibited sex differences in spatial affinity for different bathymetric habitats. Females (n = 10) were consistently localized along the northern slope of the Great Bahama Canyon in an area where the benthos fell within their dive range. The shape of this distribution pattern also appears to correspond closely with the gradual shoaling of bathymetric topography from east to west along this slope. By contrast, sub-adult males (n = 16) that were typically encountered solitarily or in small bachelor groups were found to range widely over a variety of bathymetric habitats that were both shallower and deeper than their maximum dive depth (1344 m). Finally, the dive depths of *Z*. *cavirostris* showed a relatively high correlation with bottom depths (ρ = 0.62), across a wide range of bathymetric depths occupied by this species (90% central quantile: 851.2- 2247m). All other species (*e*.*g*., *P*. *electra*, *G*. *macrorhynchus*, *P*. *macrocephalus*, and *M*. *densirostris*) showed low overall correlations of dive depths with bottom depths (ρ = 0.16–0.27).

## Discussion

### Dive behavior and body mass

Similar to previous research examining the relationship of dive duration with body mass [19–21], this study found an overall positive correlation. In particular, the relatively strong performance of the Noren & Williams (2000) [19] Eq 2 model among the delphinids and physeterids suggested that variation in body mass underlies an important component of dive duration and dive depth variation in some odontocetes species. The longer dive duration of *Z*. *cavirostris* (med. 1557 kg) relative to *M*. *densirostris* (med. 843 kg) also pointed to the relevance of body mass as a predictor of dive capacities. However, the comparatively low proportion of the overall variance in observed maximum dive duration (*T*_*max*_) explained by body mass (*m*) alone, suggested a need for additional covariates, especially to explain the dive durations of the two beaked whale species.

Moreover, selection on odontocete body size is likely considerably more complex than a simple optimization of dive efficiency and duration, so it is important to consider alternative hypotheses for the evolution of this trait. For example, larger body size generally reduces the per-unit-mass metabolic rate and external surface-area-to-volume ratios, fostering greater overall efficiency of energy use and thermoregulation [63,64]. Greater size may also decrease the cost of locomotion in a viscous fluid medium by reducing drag at higher Reynolds numbers [65,66]. Larger size may additionally confer the ability to capture a greater volume of prey during each dive, endure longer periods of fasting [67], and subsist on lower quality forage items through increased gastro-intestinal surface area and processing time [68]. Conversely, larger size may limit maneuverability in the pursuit of small prey [4,69,70]. Odontocete size may also have come under selection for reasons unrelated to foraging and energetic efficiency, such as minimizing predation risk [71] and mate competition [72].

### Dive behavior and myoglobin concentration

Although body mass is correlated with dive duration, it is unlikely that this variable directly determines diving ability, but instead represents a proxy for the quantity of oxygen stored in various tissue reservoirs (*e*.*g*., blood, muscle, lungs), relative to metabolic rate [20]. Tissue oxygen reservoirs also vary based on the concentrations of oxygen-storing molecules. However in this study, [Mb] accounted for only a minimal proportion of the variance in *T*_*max*_, *T*, and *Z* (S2 Appendix), and its inclusion could not be justified on the basis of *wDIC* metrics. This may have resulted from the specialist deep-diving taxa in this study all exhibiting high [Mb] values relative to epipelagic odontocetes, baleen whales (Mysticeti) and non-diving mammals [21]. This potentially reflects a ceiling on myoglobin concentrations ([Mb]) in muscle fibres due to self-adhesion when myoglobin units become more densely concentrated [21,31]. One potential evolutionary response is the genome-level modification of myoglobin peptide sequences to increase net surface charge, Z_Mb_, a property which increases the repulsion and decreases the adhesion of adjacent myoglobin units [21]. These authors noted elevated Z_Mb_ within the ziphiids (Z_Mb_ = 4.80) relative to delphinids (Z_Mb_ = 4.03), physeterids (Z_Mb_ = 4.15), and kogiids (Z_Mb_ = 4.24). Yet despite this elevated Z_Mb_ value, empirical measurements of bulk epaxial muscle [Mb] among ziphiids did not appear to be exceptional relative to other specialist deep-diving odontocete taxa [19,73,74]. This suggests a need for additional explanatory variables to account for the extended dive durations and deep dive depths of ziphiids with respect to their body masses.

### Dive behavior and aerobic dive limits

Utilizing anaerobic metabolism after depleting muscle oxygen reservoirs represents a potential strategy that could extend dive durations and enable efficient access to bathypelagic prey resources despite the relatively small body masses of the beaked whales [7–9,33]. Tyack *et al*. (2006) [33] derived Calculated Aerobic Dive Limit (cADL) [22] values of 25 min for *M*. *densirostris* and 33 min for *Z*. *cavirostris*, which were substantially lower than our observed mean foraging dive duration of *M*. *densirostris* and *Z*. *cavirostris* (46.2 min and 65.3 min, respectively).

$$cADL=\frac{body\;O_{2}\;reservoirs}{diving\;metabolic\;rate}$$

However, notably Velten *et al*. (2013) [74] proposed cADL estimates for *M*. *densirostris* in excess of observed foraging dive duration values, based on economical diving locomotion (including substantial gliding) and several distinct histological characteristics of the beaked whales (see *Ziphiid Synthesis*). Several empirical studies of diving metabolic rates (dMR) in other diving taxa suggest that dMR generally exceeds basal metabolic rates (bMR) by a factor of ~2x [23,75]. However the dMR values used in cADL calculations by Velten *et al*. (2013) [74] were 18% and 37% lower than bMR values estimated for these species [63], calling their cADL estimates in question.

The protracted IDDI of *M*. *densirostris* and *Z*. *cavirostris* provided further evidence suggesting the extension of beaked whale dive durations beyond ADL. Tyack *et al*. (2006) [33] interpreted extended IDDI as periods of recovery from lactate debt, citing increases in lactate concentrations and extension of inter-dive recovery periods observed in *L*. *weddellii* after dives exceeding the adult (~450kg) ADL threshold (16.8 min) (Kooyman et al. 1983) [22]. Our study confirmed the patterns of extended IDDI first documented by Tyack *et al*. (2006) [33] over a more extensive dataset of time series and behavior log dive profiles from *M*. *densirostris* (695.91 hr) and *Z*. *cavirostris* (365.65 hr). The inference from the GLM and PGLMM models of *T*_*max*_, *T*, and *Z* (S2 Appendix), also strongly pointed to the importance of IDDI as a key variable in explaining the dive duration variability among our study species, and our PGLS analysis extended this inference to a wider range of odontocete taxa.

However, one important caveat to consider is that both *M*. *densirostris* and *Z*. *cavirostris* exhibited very infrequent (≤1.46% of IDDI), but notable, sequential deep foraging dives with an intervening IDDI of <10 min. Although exceedingly rare, these short IDDI raised the question of whether prolonged IDDI represent an absolute and immediate necessity to metabolize accumulated lactate. This question hinges on whether ziphiids are diving at or near their physiological capacity as proposed by Tyack *et al*. (2006) [33], or whether they are able to pursue multiple extended dives in short succession by tolerating and buffering accumulated lactic acid and metabolizing it at a later time [76]. Comparison of buffering capacities of muscle and blood tissues between ziphiids and other odontocetes represents an intriguing line of research.

The temporal partitioning of metabolically costly food digestion from oxygen-constrained food acquisition [77] as well as social behaviors [78] represent potential alternative explanations for the extension of IDDI among beaked whales. However, it is intriguing that neither the delphinids nor physeterids required similar pauses between dives. The inclusion of IDDI in GLM, PGLMM, and PGLS models, and then extension of dive durations beyond the cADL calculated by Tyack *et al*. (2006) [33], both point to the use of anaerobic metabolism among ziphiids to extend the duration of most deep dives. This may allow ziphiids to access prey resources that would otherwise lie beyond the economical reach of their dive capacities given their relatively small body sizes.

Our data demonstrated that the extended IDDIs of ziphiids have important consequences in terms of the proportion of time available for foraging relative to other species. After accounting for differences in commuting times necessary to reach different depths, the ziphiids spent considerably less time at target foraging depths relative to other sympatric species (max. 34–37% vs. 72–89% respectively). This discrepancy in foraging time warrants some consideration of the general ecological context of odontocete diving.

### Ecological context

Overall, the extinction of photosynthetically active light below the euphotic zone [79] and the remineralization of sinking particulate organic matter (POM) lead to roughly exponential declines in trophic inputs and calories available in food webs with depth [80,81]. An important exception is the vertically migratory component of the deep-scattering layer (DSL) [17,82,83], which constitutes an important source of prey for many odontocetes [84–86]. With increasing depth, many meso- and bathypelagic fishes, cephalopods, and crustaceans tend to increase in sedentism and exhibit more limited locomotion relative to epipelagic counterparts [17]. Deeper dwelling organisms also tend to increase in water content and decrease in protein content with depth [14–16]. Finally at the benthos, settlement and resulting concentration of sinking POM together support a larger community of benthic boundary layer (BBL) consumers than would be found in mid-waters at comparable depths [87]. Thus the density, caloric value, and mobility of prey likely all vary substantially in the vertical and temporal dimensions of the habitats in which our study species forage.

### Ziphiid synthesis

Dive duration, IDDI, and dive depth documented for the tagged beaked whales in this study suggested that these species relied on a comparatively inefficient strategy of anaerobic respiration to access lower-mesopelagic and bathypelagic prey layers. Cephalopod, fish, and crustacean prey at these depths may be less abundant and potentially less nutritious relative to shallower prey, but are also likely to be less capable of evasion and beaked whales likely face fewer competitors at these depths. The balance of diving for sufficiently long periods to energetically profit from foraging dives while recouping the costs of commuting appears to have been accomplished while maintaining relatively small body masses. Smaller mass may be particularly important in these deeper habitats, since the metabolic demands of a larger body mass may be challenging to support given the likely caloric-limitations of lower meso- and bathypelagic food webs [81].

Importantly, this information points to a particular vulnerability of beaked whales to chronic acoustic disturbances from naval sonar, seismic mapping, and even vessel engine noise. These species likely subsist on relatively nutritionally marginal prey resources [16] and also invest a comparatively high proportion of their time and energy into each foraging dive, relative to other deep-diving cetaceans. The interruption of normal foraging behaviors, that has been observed during experimental and real-time sonar disturbances [39,88,89] may therefore have a greater impact on stress, reproduction, and potentially survival of these beaked whales than previously described [90].

Regular reliance on anaerobic respiration may also account for several intriguing findings in previous studies [21,74,91] where high proportions of Type II glycolytic muscle fibers were found in the epaxial muscles of *Mesoplodon* spp. and *Z*. *cavirostris* relative to other deep diving species (*e*.*g*., *G*. *macrorhychus*). Velten *et al*. (2013) proposed that this elevated volume of Type II glycolytic fibers (76–83% by cross-sectional area in *Mesoplodon* spp.) might represent “a metabolically inexpensive oxygen store within the muscle for use of less abundant Type I fibers.” We suggest that this finding might alternatively represent an adaptation enabling sustained anaerobic muscular function. Additionally, the proportional rarity of aerobic Type I fibers, which typically have higher [*Mb*] than glycolytic Type II fibers [92–94], might explain the relatively high net surface charge (Z_Mb_ = 4.80) but overall similarity of bulk [*Mb*] between the beaked whales and other deep diving species [19,21,60,74]. This elevated Z_Mb_ might allow the beaked whales to more tightly space myoglobin units within proportionally rare Type I fibers.

In addition to the likely extension of foraging dives beyond ADL, ziphiids also exhibit a range of other metabolically economical adaptations that may allow further extension of dive durations. These include differences in the proportion by weight of tissue that incur high metabolic maintenance costs (*e*.*g*., brain and visceral tissues) versus low-cost tissues (*e*.*g*., adipose, bone, and inactive muscles) when compared to other deep-diving species [95]. Additionally, *Mesoplodon* spp. and *Z*. *cavirostris* exhibit large-diameter muscle fibres, which reduce cellular surface-area-to-volume ratios and may thus minimize the metabolic demand of the active ion pumps needed to maintain muscle fibre membrane potential [74,96,97]. Finally, these species exhibit considerably lower mitochondrial densities in muscle tissues relative to *G*. *macrorhynchus* [74,91]. These metabolic adaptations likely contribute to longer, more efficient dives and are also consistent with the more limited evasive capacities of target prey species.

### Physeterid synthesis

In contrast to the beaked whales, the large body mass of sperm whales may enable this species considerable flexibility to aerobically access prey resources over a wide range of depths. This flexibility is reflected in the wide range of reported prey from stomach contents [84–86], and also reports from various locations of diving/foraging activity in bathypelagic [98], mesopelagic [3] and epipelagic [99] depths. In our study area, *P*. *macrocephalus* appeared to exploit central and lower mesopelagic layers, in which prey were likely moderately more abundant and potentially more nutritionally rewarding, compared to the deeper habitats occupied by the beaked whales. It is likely that large body masses also required large caloric inputs, potentially limiting the ability of sperm whales to sustain themselves on sparser food webs at greater depths. Finally, larger size, and also potentially positive buoyancy [69], may limit the ability of sperm whales to pursue more maneuverable and evasive prey at shallower depths.

### Delphinid synthesis

We hypothesize that the smaller body masses and other traits such as high mitochondrial densities [60,74,91], may limit the duration of *G*. *macrorhynchus* and *P*. *electra* dives, and thus their access to lower meso- and bathypelagic niches. However, these traits may also enable them to pursue more evasive and potentially more nutritious components of the DSL [16]. The size differences between *G*. *macrorhynchus* and *P*. *electra* may reflect the utilization of different DSL components. Specifically, *P*. *electra* did not appear to undertake any daytime deep dives (Fig 7), indicating that energetically advantageous prey may be too deep during daylight periods and only become accessible as these prey migrated upward at night. In contrast, *G*. *macrorhynchus* pursued a mixed strategy similar to the pattern described by Aguilar de Soto *et al*. (2008) [4]. This pattern consisted of less frequent, deeper (maximum 984 m; Figs 2 and 8), and more highly aerobic daytime sprint-pursuit dives [4], and more frequent and shallower nighttime dives that potentially overlapped the prey pursued by *P*. *electra* (Fig 1).

### Spatial and temporal habitat use patterns

Examination of movement tracks with regard to bottom topography in the Great Bahama Canyon yielded additional insights into species’ differences in foraging habitat. The telemetry information from our five study species suggested two general distribution patterns. Species exhibiting the first pattern were the shallower and more diurnally variable divers *P*. *electra* and *G*. *macrorhynchus*, as well as sub-adult male *P*. *macrocephalus*, which ranged widely over habitats with a variety of bathymetric depths, including habitats substantially deeper than their observed dive depth ranges. In contrast, *M*. *densirostris*, *Z*. *cavirostris*, and adult female *P*. *macrocephalus* exhibited a second, more localized distribution pattern that was more tightly correlated with the spatial distribution of benthic habitats accessible within their respective dive ranges. This suggested that these deeper divers might have been targeting BBL prey resources, while the shallower divers were potentially targeting more widely distributed mid-water DSL prey resources.

Benthic echoes recorded by digital acoustic recording tags (DTAG) deployed on *M*. *densirostris*, have already directly shown that this species often maintains close proximity to the benthos during a portion of foraging dives [5]. Although our telemetry data is more circumstantial than the direct evidence provided by Arranz *et al*. (2011) [5], it also suggested that *M*. *densirostris*, *Z*. *cavirostris*, and *P*. *macrocephalus*, to varying degrees, interacted with prey layers tied geographically to benthic habitats. A specific example was the close match between the wedge-shaped westward spreading of tracks belonging to female *P*. *macrocephalus* and the gradual shoaling of benthic topography into the dive range of female *P*. *macrocephalus* along the northern slope of the Great Bahama Canyon. Furthermore the correlation of *Z*. *cavirostris* dive depths with benthic depths at estimated dive locations also circumstantially supported a hypothesis of BBL prey use. Intriguingly *M*. *densirostris* exhibited some inter-individual variation in spatial association with the benthos, which may reflect the flexibility in foraging strategy (i.e., switching between mid-water lower mesopelagic prey and BBL prey) documented by Arranz *et al*. (2011) [5]. As further evidence of behavioral complexity, *M*. *densirostris* also exhibited shallower daytime and deeper nighttime dives, which may indicate either the pursuit of prey that undertook a reverse diel vertical migration, or a diurnal switch between mid-water and lower meso- and benthopelagic resources as shown by Arranz *et al*. (2011) [5].

### Conclusions

In conclusion, this study confirmed that body mass is an important correlate of dive durations in toothed whales and is integral to their ability to access prey at different depths. However, substantial evidence also supported the hypothesis of Tyack *et al*. (2006) [33] that the beaked whales employ a mix of aerobic and anaerobic respiration to extend dive duration during deep foraging dives. We suggest that this represents an alternative strategy for accessing deeper prey resources without growing large, and that this strategy is likely related to limited prey availability below mesopelagic deep scattering layers, where the relative importance of benthopelagic prey also increases. Our study highlights likely evolutionary trade-offs that have shaped the bodies and behaviors of deep-diving toothed whales. Finally, this study underlines important variations in vulnerability and exposure of different odontocetes to anthropogenic impacts, particularly acoustic disturbances.

## Supporting information

S1 Appendix Estimates of body mass and myoglobin concentration for different species, sexes and age classes of odontocete. (PDF)

S2 Appendix Phylogenetic Generalized Linear Mixed Models of dive duration and dive depth. (PDF)

## References

[1] Mayo CA, Marx MK. Surface foraging behaviour of the North Atlantic right whale, *Eubalaena glacialis*, and associated zooplankton characteristics. Can J Zool. 1990;68: 2214–2220.

[2] Benoit-Bird KJ, Würsig B, Mfadden CJ. Dusky dolphin (*Lagenorhynchus obscurus*) foraging in two different habitats: active acoustic detection of dolphins and their prey. Mar Mammal Sci. 2004;20: 215–231.

[3] Watwood SL, Miller PJ, Johnson M, Madsen PT, Tyack PL. Deep-diving foraging behaviour of sperm whales (*Physeter macrocephalus*). J Anim Ecol. 2006;75: 814–825. doi: 10.1111/j.1365-2656.2006.01101.x 16689963 10.1111/j.1365-2656.2006.01101.x

[4] Aguilar Soto N, Johnson MP, Madsen PT, Díaz F, Domínguez I, Brito A, et al Cheetahs of the deep sea: deep foraging sprints in short-finned pilot whales off Tenerife (Canary Islands). J Anim Ecol. 2008;77: 936–947. doi: 10.1111/j.1365-2656.2008.01393.x 18444999 10.1111/j.1365-2656.2008.01393.x

[5] Arranz P, De Soto NA, Madsen PT, Brito A, Bordes F, Johnson MP. Following a foraging fish-finder: Diel habitat use of Blainville’s beaked whales revealed by echolocation. PloS One. 2011;6: e28353 doi: 10.1371/journal.pone.0028353 22163295 10.1371/journal.pone.0028353 PMC3233560

[6] Houston AI, Carbone C. The optimal allocation of time during the diving cycle. Behav Ecol. 1992;3: 255–265.

[7] Carbone C, Houston AI. The optimal allocation of time over the dive cycle: an approach based on aerobic and anaerobic respiration. Anim Behav. 1996;51: 1247–1255.

[8] Mori Y. The optimal allocation of time and respiratory metabolism over the dive cycle. Behav Ecol. 1999;10: 155–160.

[9] Mori Y. Optimal diving behaviour for foraging in relation to body size. J Evol Biol. 2002;15: 269–276.

[10] Costa DP, Gales NJ, Goebel ME. Aerobic dive limit: how often does it occur in nature? Comp Biochem Physiol A Mol Integr Physiol. 2001;129: 771–783. 11440864 10.1016/s1095-6433(01)00346-4

[11] Georges J-Y, Tremblay Y, Guinet C. Seasonal diving behaviour in lactating subantarctic fur seals on Amsterdam Island. Polar Biol. 2000;23: 59–69.

[12] Johnson MW. Sound as a tool in marine ecology, from data on biological noises and the deep scattering layer. J Mar Res. 1948;7: 443–458.

[13] Marshall NB. Systematic and biological studies of the macrourid fishes (Anacanthini-Teleostii) In: Deep Sea Research and Oceanographic Abstracts. Amsterdam: Elsevier; 1965 pp. 299–322.

[14] Stickney DG, Torres JJ. Proximate composition and energy content of mesopelagic fishes from the eastern Gulf of Mexico. Mar Biol. 1989;103: 13–24.

[15] Bailey TG, Robison BH. Food availability as a selective factor on the chemical compositions of midwater fishes in the eastern North Pacific. Mar Biol. 1986;91: 131–141.

[16] Childress JJ, Nygaard MH. The chemical composition of midwater fishes as a function of depth of occurence off southern California In: Deep Sea Research and Oceanographic Abstracts. Amsterdam: Elsevier; 1973 pp. 1093–1109.

[17] Childress JJ. Are there physiological and biochemical adaptations of metabolism in deep-sea animals? Trends Ecol Evol. 1995;10: 30–36. 21236941 10.1016/s0169-5347(00)88957-0

[18] Waring GT, Hamazaki T, Sheehan D, Wood G, Baker S. Characterization of beaked whale (Ziphiidae) and sperm whale (*Physeter macrocephalus*) summer habitat in shelf-edge and deeper waters off the northeast US. Mar Mammal Sci. 2001;17: 703–717.

[19] Noren SR, Williams TM. Body size and skeletal muscle myoglobin of cetaceans: adaptations for maximizing dive duration. Comp Biochem Physiol A Mol Integr Physiol. 2000;126: 181–191. 10936758 10.1016/s1095-6433(00)00182-3

[20] Halsey LG, Butler PJ, Blackburn TM. A phylogenetic analysis of the allometry of diving. Am Nat. 2006;167: 276–287. doi: 10.1086/499439 16670986 10.1086/499439

[21] Mirceta S, Signore AV, Burns JM, Cossins AR, Campbell KL, Berenbrink M. Evolution of mammalian diving capacity traced by myoglobin net surface charge. Science. 2013;340: 1234192 doi: 10.1126/science.1234192 23766330 10.1126/science.1234192

[22] Kooyman GL, Castellini MA, Davis RW, Maue RA. Aerobic diving limits of immature Weddell seals. J Comp Physiol [B]. 1983;151: 171–174.

[23] Castellini MA, Kooyman GL, Ponganis PJ. Metabolic rates of freely diving Weddell seals: correlations with oxygen stores, swim velocity and diving duration. J Exp Biol. 1992;165: 181–194. 1588250 10.1242/jeb.165.1.181

[24] Schreer JF, Kovacs KM. Allometry of diving capacity in air-breathing vertebrates. Can J Zool. 1997;75: 339–358.

[25] Butler PJ, Jones DR. The comparative physiology of diving in vertebrates. Adv Comp Physiol Biochem. 1982;8: 179–364. 6753521 10.1016/b978-0-12-011508-2.50012-5

[26] Lomolino MV. Body size of mammals on islands: the island rule reexamined. Am Nat. 1985;125: 310–316.

[27] McClain CR, Boyer AG, Rosenberg G. The island rule and the evolution of body size in the deep sea. J Biogeogr. 2006;33: 1578–1584.

[28] Snyder GK. Respiratory adaptations in diving mammals. Respir Physiol. 1983;54: 269–294. 6369460 10.1016/0034-5687(83)90072-5

[29] Ponganis PJ, Meir JU, Williams CL. In pursuit of Irving and Scholander: a review of oxygen store management in seals and penguins. J Exp Biol. 2011;214: 3325–3339. doi: 10.1242/jeb.031252 21957096 10.1242/jeb.031252

[30] Wells RE Jr, Merrill EW. Influence of flow properties of blood upon viscosity-hematocrit relationships. J Clin Invest. 1962;41: 1591 doi: 10.1172/JCI104617 14040228 10.1172/JCI104617 PMC291077

[31] Lawrence MS, Phillips KJ, Liu DR. Supercharging proteins can impart unusual resilience. J Am Chem Soc. 2007;129: 10110–10112. doi: 10.1021/ja071641y 17665911 10.1021/ja071641y PMC2820565

[32] Kooyman GL, Wahrenbrock EA, Castellini MA, Davis RW, Sinnett EE. Aerobic and anaerobic metabolism during voluntary diving in Weddell seals: evidence of preferred pathways from blood chemistry and behavior. J Comp Physiol [B]. 1980;138: 335–346.

[33] Tyack PL, Johnson M, Soto NA, Sturlese A, Madsen PT. Extreme diving of beaked whales. J Exp Biol. 2006;209: 4238–4253. doi: 10.1242/jeb.02505 17050839 10.1242/jeb.02505

[34] Castellini MA, Somero GN. Buffering capacity of vertebrate muscle: correlations with potentials for anaerobic function. J Comp Physiol. 1981;143: 191–198.

[35] Gillespie D, Dunn C, Gordon J, Claridge D, Embling C, Boyd I. Field recordings of Gervais’ beaked whales *Mesoplodon europaeus* from the Bahamas. J Acoust Soc Am. 2009;125: 3428–3433. doi: 10.1121/1.3110832 19425681 10.1121/1.3110832

[36] DiMarzio N, Moretti D, Ward J, Morrissey R, Jarvis S, Izzi AM, et al Passive acoustic measurement of dive vocal behavior and group size of Blainville’s beaked whale (*Mesoplodon densirostris*) in the Tongue of the Ocean (TOTO). Can Acoust. 2008;36: 166–172.

[37] Andrews RD, Pitman RL, Ballance LT. Satellite tracking reveals distinct movement patterns for Type B and Type C killer whales in the southern Ross Sea, Antarctica. Polar Biol. 2008;31: 1461–1468.

[38] Schorr GS, Falcone EA, Moretti DJ, Andrews RD. First long-term behavioral records from Cuvier’s beaked whales (*Ziphius cavirostris*) reveal record-breaking dives. PLoS One. 2014;9: e92633 doi: 10.1371/journal.pone.0092633 24670984 10.1371/journal.pone.0092633 PMC3966784

[39] Tyack PL, Zimmer WM, Moretti D, Southall BL, Claridge DE, Durban JW, et al Beaked whales respond to simulated and actual navy sonar. PloS One. 2011;6: e17009 doi: 10.1371/journal.pone.0017009 21423729 10.1371/journal.pone.0017009 PMC3056662

[40] Hooker SK, Baird RW. Diving and ranging behaviour of odontocetes: a methodological review and critique. Mammal Rev. 2001;31: 81–105.

[41] Rosel PE. PCR-based sex determination in Odontocete cetaceans. Conserv Genet. 2003;4: 647–649.

[42] Morin PA, Nestler A, Rubio-Cisneros NT, Robertson KM, Mesnick SL. Interfamilial characterization of a region of the ZFX and ZFY genes facilitates sex determination in cetaceans and other mammals. Mol Ecol. 2005;14: 3275–3286. doi: 10.1111/j.1365-294X.2005.02651.x 16101791 10.1111/j.1365-294X.2005.02651.x

[43] Johnson DS, London JM, Lea M-A, Durban JW. Continuous-time correlated random walk model for animal telemetry data. Ecology. 2008;89: 1208–1215. 18543615 10.1890/07-1032.1

[44] Johnson DS. *crawl*: fit continuous-time correlated random walk models to animal movement data. R package version 1.4–1 Vienna: R Foundation for Statistical Computing; 2013.

[45] McClintock BT, London JM, Cameron MF, Boveng PL. Modelling animal movement using the Argos satellite telemetry location error ellipse. Methods Ecol Evol. 2015;6: 266–277.

[46] Hijmans RJ, van Etten J. *raster*: Geographic analysis and modeling with raster data. R package version 2.0–12 Vienna: R Foundation for Statistical Computing; 2012.

[47] Joyce TW, Durban JW, Fearnbach H, Claridge D, Ballance LT. Use of time-at-temperature data to describe dive behavior in five species of sympatric deep-diving toothed whales. Mar Mammal Sci. 2016;32: 1044–1071.

[48] Miller PJ, Johnson MP, Tyack PL. Sperm whale behaviour indicates the use of echolocation click buzzes “creaks” in prey capture. Proc R Soc Lond B Biol Sci. 2004;271: 2239–2247. 10.1098/rspb.2004.2863 PMC1691849 15539349

[49] de Villemereuil P, Nakagawa S. General quantitative genetic methods for comparative biology In: Garamszegi LZ, editor. Modern Phylogenetic Comparative Methods and Their Application in Evolutionary Biology. Berlin: Springer; 2014 pp. 287–303.

[50] McGowen MR, Spaulding M, Gatesy J. Divergence date estimation and a comprehensive molecular tree of extant cetaceans. Mol Phylogenet Evol. 2009;53: 891–906. doi: 10.1016/j.ympev.2009.08.018 19699809 10.1016/j.ympev.2009.08.018

[51] Zuur AF, Ieno EN, Walker NJ, Saveliev AA, Smith GM. Mixed effects models and extensions in ecology with R New York: Springer New York; 2009.

[52] Burnham KP, Anderson DR. Model Selection and Multimodel Inference. New York: Springer New York; 2004.

[53] Grafen A. The phylogenetic regression. Philos Trans R Soc Lond B Biol Sci. 1989;326: 119–157. 2575770 10.1098/rstb.1989.0106

[54] Martin AR, Smith TG. Strategy and capability of wild belugas, *Delphinapterus leucas*, during deep, benthic diving. Can J Zool. 1999;77: 1783–1793.

[55] Laidre KL, Heide-Jørgensen MP, Dietz R. Diving behaviour of narwhals (*Monodon monoceros*) at two coastal localities in the Canadian High Arctic. Can J Zool. 2002;80: 624–635.

[56] Westgate AJ, Head AJ, Berggren P, Koopman HN, Gaskin DE. Diving behaviour of harbour porpoises, *Phocoena phocoena*. Can J Fish Aquat Sci. 1995;52: 1064–1073.

[57] Reisinger RR, Keith M, Andrews RD, de Bruyn PJN. Movement and diving of killer whales (*Orcinus orca*) at a Southern Ocean archipelago. J Exp Mar Biol Ecol. 2015;473: 90–102.

[58] Baird RW, Ligon AD, Hooker SK, Gorgone AM. Subsurface and nighttime behaviour of pantropical spotted dolphins in Hawai’i. Can J Zool. 2001;79: 988–996.

[59] Scott M, Hohn A, Westgate A, Nicolas J, Whitaker B, Campbell W. A note on the release and tracking of a rehabilitated pygmy sperm whale (*Kogia breviceps*). J Cetacean Res Manage. 2001;3: 87–94.

[60] Kielhorn CE, Dillaman RM, Kinsey ST, McLellan WA, Gay DM, Dearolf JL, et al Locomotor muscle profile of a deep (*Kogia breviceps*) versus shallow (*Tursiops truncatus*) diving cetacean. J Morphol. 2013;274: 663–675. doi: 10.1002/jmor.20124 23355398 10.1002/jmor.20124

[61] Hooker SK, Baird RW. Deep–diving behaviour of the northern bottlenose whale, *Hyperoodon ampullatus* (Cetacea: Ziphiidae). Proc R Soc Lond B Biol Sci. 1999;266: 671–676.

[62] Nakagawa S, Schielzeth H. A general and simple method for obtaining R^2^ from generalized linear mixed-effects models. Methods Ecol Evol. 2013;4: 133–142.

[63] Kleiber M. Metabolic turnover rate: a physiological meaning of the metabolic rate per unit body weight. J Theor Biol. 1975;53: 199–204. 1195755 10.1016/0022-5193(75)90110-1

[64] Hayssen V, Lacy RC. Basal metabolic rates in mammals: taxonomic differences in the allometry of BMR and body mass. Comp Biochem Physiol A Physiol. 1985;81: 741–754. 10.1016/0300-9629(85)90904-1 2863065

[65] Lang TG. Hydrodynamic analysis of cetacean performance In: Norris KS, editor. Whales Porpoises Dolphins. Berkeley: University of California Press 1966; pp. 410–432.

[66] Sato K, Watanuki Y, Takahashi A, Miller PJ, Tanaka H, Kawabe R, et al Stroke frequency, but not swimming speed, is related to body size in free-ranging seabirds, pinnipeds and cetaceans. Proc R Soc Lond B Biol Sci. 2007;274: 471–477. 10.1098/rspb.2006.0005 PMC1766382 17476766

[67] Lindstedt SL, Boyce MS. Seasonality, fasting endurance, and body size in mammals. Am Nat. 1985;125: 873–878.

[68] Demment MW, Van Soest PJ. A nutritional explanation for body-size patterns of ruminant and nonruminant herbivores. Am Nat. 1985; 5: 641–672.

[69] Miller PJ, Johnson MP, Tyack PL, Terray EA. Swimming gaits, passive drag and buoyancy of diving sperm whales *Physeter macrocephalus*. J Exp Biol. 2004;207: 1953–1967. 15107448 10.1242/jeb.00993

[70] Aoki K, Amano M, Mori K, Kourogi A, Kubodera T, Miyazaki N. Active hunting by deep-diving sperm whales: 3D dive profiles and maneuvers during bursts of speed. Mar Ecol Prog Ser. 2012;444: 289–301.

[71] Sinclair ARE, Mduma S, Brashares JS. Patterns of predation in a diverse predator–prey system. Nature. 2003;425: 288–290. doi: 10.1038/nature01934 13679915 10.1038/nature01934

[72] Clutton-Brock TH. Reproductive success: studies of individual variation in contrasting breeding systems Chicago: University of Chicago Press; 1988.

[73] Sharp JG, Marsh BB. Whalemeat: Production and Preservation. London: UK Department of Scientific and Industrial Research; 1953.

[74] Velten BP, Dillaman RM, Kinsey ST, McLellan WA, Pabst DA. Novel locomotor muscle design in extreme deep-diving whales. J Exp Biol. 2013;216: 1862–1871. doi: 10.1242/jeb.081323 23393275 10.1242/jeb.081323

[75] Ponganis PJ, Kooyman GL, Castellini MA. Determinants of the aerobic dive limit of Weddell seals: analysis of diving metabolic rates, postdive end tidal pO₂’s, and blood and muscle oxygen stores. Physiol Zool. 1993;5: 732–749.

[76] Hazen EL, Friedlaender AS, Goldbogen JA. Blue whales (*Balaenoptera musculus*) optimize foraging efficiency by balancing oxygen use and energy gain as a function of prey density. Sci Adv. 2015;1: e1500469 doi: 10.1126/sciadv.1500469 26601290 10.1126/sciadv.1500469 PMC4646804

[77] Rosen DA, Gerlinsky CD, Trites AW. Evidence of partial deferment of digestion during diving in Steller sea lions (*Eumetopias jubatus*). J Exp Mar Biol Ecol. 2015;469: 93–97.

[78] Sparling CE, Fedak MA, Thompson D. Eat now, pay later? Evidence of deferred food-processing costs in diving seals. Biol Lett. 2007;3: 95–99. 10.1098/rsbl.2006.0566 PMC2373816 17443975

[79] Jerlov NG. Optical oceanography Amsterdam: Elsevier; 1968.

[80] Martin JH, Knauer GA, Karl DM, Broenkow WW. VERTEX: carbon cycling in the northeast Pacific. Deep Sea Res Part Oceanogr Res Pap. 1987;34: 267–285.

[81] Gage JD, Tyler PA. Deep-sea biology: a natural history of organisms at the deep-sea floor Cambridge, UK: Cambridge University Press; 1991.

[82] Barham EG. Deep scattering layer migration and composition: observations from a diving saucer. Science. 1966;151: 1399–1403. doi: 10.1126/science.151.3716.1399 17817303 10.1126/science.151.3716.1399

[83] Ohman MD, Frost BW, Cohen EB. Reverse diel vertical migration: an escape from invertebrate predators. Science. 1983;220: 1404–1407. doi: 10.1126/science.220.4604.1404 17730658 10.1126/science.220.4604.1404

[84] Evans K, Hindell MA. The diet of sperm whales (*Physeter macrocephalus*) in southern Australian waters. ICES J Mar Sci J Cons. 2004;61: 1313–1329.

[85] Smith SC, Whitehead H. The diet of Galapagos sperm whales *Physeter macrocephalus* as indicated by fecal sample analysis. Mar Mammal Sci. 2000;16: 315–325.

[86] Clarke MR. Cephalopods as prey. III. Cetaceans. Philos Trans R Soc B Biol Sci. 1996;351: 1053–1065.

[87] Angel MV, Boxshall GA. Life in the benthic boundary layer: connections to the mid-water and sea floor [and Discussion]. Philos Trans R Soc Lond Math Phys Eng Sci. 1990;331: 15–28.

[88] McCarthy E, Moretti D, Thomas L, DiMarzio N, Morrissey R, Jarvis S, et al Changes in spatial and temporal distribution and vocal behavior of Blainville’s beaked whales (*Mesoplodon densirostris*) during multiship exercises with mid-frequency sonar. Mar Mammal Sci. 2011;27: E206–E226.

[89] Moretti D, Thomas L, Marques T, Harwood J, Dilley A, Neales B, et al A risk function for behavioral disruption of Blainville’s beaked whales (*Mesoplodon densirostris*) from mid-frequency active sonar. PloS One. 2014;9: e85064 doi: 10.1371/journal.pone.0085064 24465477 10.1371/journal.pone.0085064 PMC3897412

[90] New LF, Moretti DJ, Hooker SK, Costa DP, Simmons SE. Using energetic models to investigate the survival and reproduction of beaked whales (family Ziphiidae). PloS One. 2013;8: e68725 doi: 10.1371/journal.pone.0068725 23874737 10.1371/journal.pone.0068725 PMC3714291

[91] Sierra E, Fernández A, de los Monteros AE, Díaz-Delgado J, de Quirós YB, García-Álvarez N, et al Comparative histology of muscle in free ranging cetaceans: shallow versus deep diving species. Sci Rep. 2015;5: 15909 doi: 10.1038/srep15909 26514564 10.1038/srep15909 PMC4626863

[92] Drews GA, Engel WK. An attempt at histochemical localization of myoglobin in skeletal muscle by the benzidine-peroxidase reaction. J Histochem Cytochem. 1961;9: 206–207. doi: 10.1177/9.2.206 13887823 10.1177/9.2.206

[93] Peter JB, Barnard RJ, Edgerton VR, Gillespie CA, Stempel KE. Metabolic profiles of three fiber types of skeletal muscle in guinea pigs and rabbits. Biochemistry. 1972;11: 2627–2633. 4261555 10.1021/bi00764a013

[94] Nemeth PM, Lowry OH. Myoglobin levels in individual human skeletal muscle fibers of different types. J Histochem Cytochem. 1984;32: 1211–1216. doi: 10.1177/32.11.6491255 6491255 10.1177/32.11.6491255

[95] Pabst DA, McLellan WA, Rommel SA. How to Build a Deep Diver: The Extreme Morphology of Mesoplodonts. Integr Comp Biol. 2016;56: 1337 doi: 10.1093/icb/icw126 27940620 10.1093/icb/icw126

[96] Johnston IA, Abercromby M, Vieira VL, Sigursteindóttir RJ, Kristjánsson BK, Sibthorpe D, et al Rapid evolution of muscle fibre number in post-glacial populations of Arctic charr *Salvelinus alpinus*. J Exp Biol. 2004;207: 4343–4360. doi: 10.1242/jeb.01292 15557021 10.1242/jeb.01292

[97] Jimenez AG, Dasika SK, Locke BR, Kinsey ST. An evaluation of muscle maintenance costs during fiber hypertrophy in the lobster *Homarus americanus*: are larger muscle fibers cheaper to maintain? J Exp Biol. 2011;214: 3688–3697. doi: 10.1242/jeb.060301 21993799 10.1242/jeb.060301

[98] Watkins WA, Daher MA, Fristrup KM, Howald TJ, Sciara D, Notarbartolo G. Sperm whales tagged with transponders and tracked underwater by sonar. Mar Mammal Sci. 1993;9: 55–67.

[99] Teloni V, Mark JP, Patrick MJ, Peter MT. Shallow food for deep divers: Dynamic foraging behavior of male sperm whales in a high latitude habitat. J Exp Mar Biol Ecol. 2008;354: 119–131.

