# Supplementary material for: Physiological, morphological, and ecological tradeoffs influence vertical habitat use of deep-diving toothed-whales in the Bahamas
Source: PLoS One. 2017 Oct 11;12(10):e0185113. doi: 10.1371/journal.pone.0185113 (PMC5636075; doi:10.1371/journal.pone.0185113)
Supplement: S1 Appendix — (PDF) [file pone.0185113.s001.pdf]

Physiological, morphological, and ecological tradeoffs influence divergent vertical habitat use among deep-diving toothed-whales of the Bahamas

Trevor W. Joyce*, John W. Durban, Diane E. Claridge, Charlotte Dunn, Holly Fearnbach, Kim Parsons, Russel Andrews, Lisa T. Ballance

*Corresponding author: trevorwjoyce@gmail.com

S1 Appendix: Estimates of body mass and myoglobin concentration for different species sexes and age classes of odontocete

The body masses of our study species were reported inconsistently across the literature, using a variety of different summary statistics (*e.g.*, maximum, mean, approximation). To standardize estimates of body mass used in the allometric analyses reported in this paper we developed estimates of median body masses in the different sexes and age classes of our study species using standard length and body mass measurements from strandings and historic whaling records from around the world. We also collected myoglobin concentration ([*Mb*]) measurements from a variety of published sources for consideration in the subsequent modeling of dive durations and dive depths.

Methods

Standard length and body mass measurements were retrieved from the National Museum of Natural History (NMNH) whale collection database (http://collections.nmnh.si.edu/search/mammals/ accessed on July 13, 2015), as well as a variety of primary literature sources not included in the NMNH database [1–5]. The mass data provided by all of these sources was relatively sparse and systematically biased towards individuals with smaller standard lengths in many species, likely due to the logistical challenges of measuring mass in animals at the scale of many cetaceans. This systematic bias presented challenges in estimating representative median masses for each species, sex, and age class based on the mass measurements alone. To overcome this challenge, we estimated power-law relationships of body mass to standard length for each species. Following the approach of Kasuya and Matsui (1984) [4], this relationship was modeled as a linear function of log-transformed body mass and standard length measurements using *lm* function from the R library *stats*. Extensive standard length measurement data from NMNH and other sources [2,3] were then used to estimate the median standard length of each species, sex, and age class and these values were then used to predict the median mass of each species, sex, and age class from the power-law relationships detailed above. Threshold lengths separating sub-adult from adult age classes in the standard length dataset were defined based on reported values [2,3] and on the minimum length at which sexual maturity characteristics, such as evidence of pregnancies and increases in gonadal mass, were described in necropsy notes.

Mean [*Mb*] values from epaxial muscles (e.g., *longissimus dorsi*) corresponding to our study species were obtained from Sharp and Marsh (1953) [6], Noren and Williams (2000) [7], Kielhorn et al. (2013) [8], Mirceta *et al.* (2013) [9], and Velten *et al.* (2013) [10]. For one study species, *Peponocephala electra*, no empirical measurements of [*Mb*] were available in the literature, consequently we estimated *P. electra* [*Mb*] from the relationship between myoglobin net surface charge (Z_{Mb}) and [*Mb*] defined in eq. 1 in Mirceta *et al.* (2013) [9]. The Z_{Mb} value used to estimate [*Mb*] for *P. electra* was reported in Supplemental Materials Table S1 from Mirceta *et al.* (2013) [9].

Results

Across the five tagged species, we compiled 175 simultaneous measurements of mass and standard length from a variety literature and unpublished sources from around the world. We compared these data with 1,912 records in which standard length alone was measured. Estimates of median body mass from power-law models relating body mass and standard length yielded adult body masses spanning nearly two orders of magnitude from *P. electra* (169.6 kg) to *P. macrocephalus* (11774.5 kg). Adult *P. macrocephalus* typically exhibited extreme sexual size dimorphism (male:female body mass ratio of 2.3), however no adult males were tagged in this study. In the modeling of dive duration and dive depth, sub-adult male body mass estimates (median 12362 kg) were used, which were only slightly greater than the body mass estimates of adult females (median 11186 kg; Table 2).

In three of our five study species mean [*Mb*] concentration values were based on measurements taken across multiple individuals (*Ziphius cavirostris*, n = 2, *Mesoplodon densirostris*, n = 2, *Globicephala macrorhynchus*, n = 6). Among these measurement derived from Noren and Williams et al. (2000) [7], and Velten et al. (2013) [10], the reported standard deviation values represented <10% of mean values, suggesting that inter-individual variation was present but relatively small. The [*Mb*] value for *P. electra* predicted from eq. 1 in Mirceta *et al.* (2013) [9], was substantially lower than the empirical mean [*Mb*] concentration value measured in the closely related *G. macrorhynchus* (Table A).

Table A. Summary of standard-length (m), mass (kg), and myoglobin (mg kg^{-1}) measurements in five species of odontocete cetaceans.

Median mass was estimated from the models presented in Fig A in S1 Appendix. Threshold standard-lengths were used to categorize measured individuals into age classes within each sex and species. The sources of [*Mb*] measurements are: 1) Mirceta *et al.* 2013, 2) Velten *et al.* 2013, 3) Noren and Williams 2000, and 4) Sharp and Marsh 1953. *note this value was not measured empirically, but estimated from eq. 1 in Mirceta *et al.* (2013).

**Species**	**Sex**	***n* Mass**	***n* Length**	**Age Class**	**Thresh. Lengths**	**Med. Length**	**Med. Mass**	**[*Mb*]**	**Mb Source**
Melon-headed whale	Male	14	33	Adult	2.45-3m	2.505	179.77	25.0*	1
Peponocephala electra				Sub-adult	1.5-2.45m	2.23	125.48		
	Female	12	45	Adult	2.3-3m	2.41	159.51		
				Sub-adult	1.5-2.3m	2.17	115.32		
Short-finned pilot whale	Male	12	93	Adult	3.95-7m	4.65	1195.4	68.2	2
Globicephala macrorhynchus				Sub-adult	3-3.95m	3.48	528.25		
	Female	32	143	Adult	2.93-7m	3.6	581.58		
				Sub-adult	2-2.93m	2.615	236.46		
Blainville's beaked whale	Male	6	52	Adult	3.5-6m	4.115	817.8	69.2	2
Mesoplodon densirostris				Sub-adult	1.5-3.5m	3.01	306.09		
	Female	9	52	Adult	3.5-6m	4.195	868.04		
				Sub-adult	1.5-3.5m	2.195	116.4		
Cuvier's beaked whales	Male	10	179	Adult	4.9-8m	5.64	1601.3	43.2	3
Ziphius cavirostris				Sub-adult	2.5-4.9m	4.01	736.03		
	Female	16	178	Adult	4.6-8m	5.5	1512.1		
				Sub-adult	2.5-4.6m	3.8	651.12		
Sperm whale	Male	51	651	Adult	12-25m	14.1	25679	70	4
Physeter macrocephalus				Sub-adult	8-12m	10.97	12362		
	Female	11	337	Adult	8.5-14m	10.6	11186		
				Sub-adult	3-8.5m	4.215	762.53		

n, number of measurements; [*Mb*], myoglobin concentration;

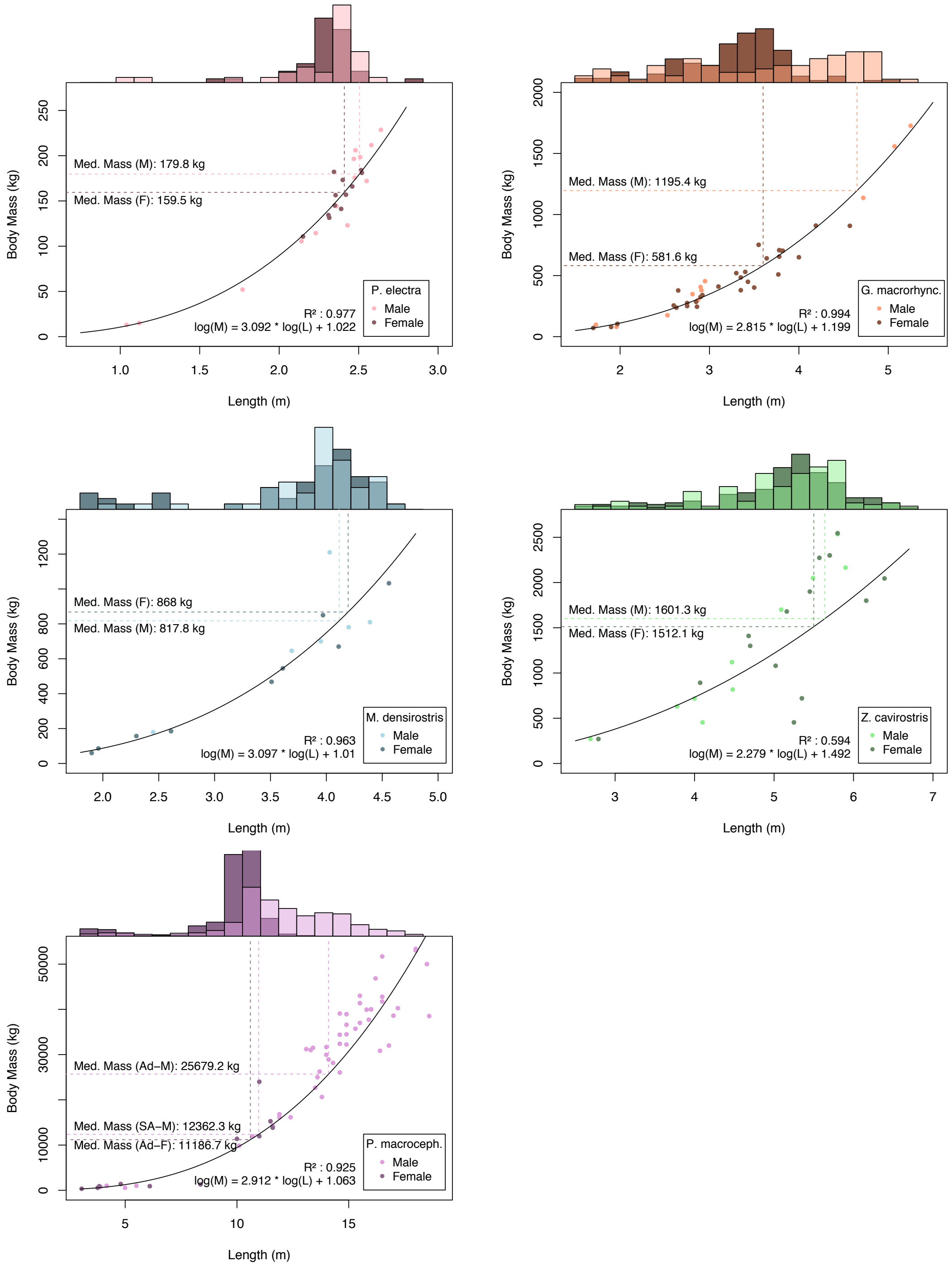

Figure A. Scatter plots showing the relationships of cetacean body mass to standard lengths in our study species.

The histograms embedded in the upper margin of each plot indicate the distributions of male (lighter shade, partially transparent) and female (darker shade) standard lengths recovered from stranding and historic whaling data. The median adult standard lengths for each species were used to estimate the median adult body masses for each species and sex using the fitted power law relationship indicated in each plot. Subadult male body mass was also estimated for *P. macrocephalus*.

References

1. Lockyer C. Body weights of some species of large whales. J Cons. 1976;36: 259–273.

2. Heyning JE. Cuvier's beaked whale Ziphius cavirostris G. Cuvier, 1823. Handb Mar Mamm. 1989;4: 289–308.

3. Mead JG. Beaked whales of the genus Mesoplodon. Handbook of marine mammals; 1989.

4. Kasuya T, Matsui S. Age determination and growth of the short-finned pilot whale off the Pacific coast of Japan. Sci Rep Whales Res Inst Tokyo. 1984;35: 57–91.

5. Miyazaki N, Fujise Y, Iwata K. Biological analysis of a mass stranding of melon-headed whales (Peponocephala electra) at Aoshima, Japan. Bull-Natl Sci Mus TOKYO Ser A. 1998;24: 31–60.

6. Sharp JG, Marsh BB. Whalemeat: Production and Preservation. UK Department of Scientific and Industrial Research, London, UK;

7. Noren SR, Williams TM. Body size and skeletal muscle myoglobin of cetaceans: adaptations for maximizing dive duration. Comp Biochem Physiol A Mol Integr Physiol. 2000;126: 181–191.

8. Kielhorn CE, Dillaman RM, Kinsey ST, McLellan WA, Mark Gay D, Dearolf JL, et al. Locomotor muscle profile of a deep (Kogia breviceps) versus shallow (Tursiops truncatus) diving cetacean. J Morphol. 2013;274: 663–675.

9. Mirceta S, Signore AV, Burns JM, Cossins AR, Campbell KL, Berenbrink M. Evolution of mammalian diving capacity traced by myoglobin net surface charge. Science. 2013;340: 1234192.

10. Velten BP, Dillaman RM, Kinsey ST, McLellan WA, Pabst DA. Novel locomotor muscle design in extreme deep-diving whales. J Exp Biol. 2013;216: 1862–1871.
