# Supplementary material for: Physiological, morphological, and ecological tradeoffs influence vertical habitat use of deep-diving toothed-whales in the Bahamas
Source: PLoS One. 2017 Oct 11;12(10):e0185113. doi: 10.1371/journal.pone.0185113 (PMC5636075; doi:10.1371/journal.pone.0185113)
Supplement: S2 Appendix — (PDF) [file pone.0185113.s002.pdf]

Physiological, morphological, and ecological tradeoffs influence divergent vertical habitat use among deep-diving toothed-whales of the Bahamas

Trevor W. Joyce*, John W. Durban, Diane E. Claridge, Charlotte Dunn, Holly Fearnbach, Kim Parsons, Russel Andrews, Lisa T. Ballance

*Corresponding author: trevorwjoyce@gmail.com

S2 Appendix. Phylogenetic Generalized Linear Mixed Models of dive duration and dive depth

Models of maximum dive duration (T_{max}), which were developed for comparability to previous modeling efforts, collapsed the variability of each individual's dive behavior to a single number, which may have inaccurately represented the typical dive behaviors of that individual or species. To account for this variability we developed additional log-log Phylogenetic Generalized Linear Mixed Models (PGLMM) [1] models of dive duration (T) and dive depth (Z) at the level of individual foraging dives.

Methods

PGLMM accounted for intraspecific non-independence of dive behaviours and phylogenetic interdependencies among species using a random effects structure that incorporated a phylogenetic tree model. We developed PGLMM using the cetacean phylogenetic tree and divergence time estimates of McGowen *et al.* (2009) [2], and three fixed-effects covariates: estimates of median body mass (m), literature-derived myoglobin concentrations ($[Mb]$), and median IDDI calculated from the behaviour log dataset. These versions of PGLMM models contained a random effect of species (SPP) that incorporated a phylogenetic correlation structure, and also random effects of individual variability (PTT) nested within species.

Bayesian posterior probability distributions for fixed-effects-only log-log (*i.e.*, power law) generalized linear models (GLM) and a log-log PGLMM models were implemented in the R function *MCMCglmm* [1]. Bayesian Markov Chain Monte Carlo (MCMC) simulations used the default Gaussian fixed-effects prior (mean = 0 and variance = 10^{10}) implemented in *MCMCglmm*, and an inverse-Gamma random-effects prior with shape and scale parameters of 0.01 [1]. MCMC simulations were repeated over 10^5 iterations, discarding a burn-in phase of 1000 iterations and applying a further thinning of 1 in every 50 iterations. In this initial phase of comparing model random-effects structures, the fixed-effects and mixed-effects log-log models were evaluated on the basis of Deviance Information Criterion (DIC) scores, and a model weighting metric, *w*DIC, that was developed based on the Akaike's weight metric (*w*AIC) from Burnham & Anderson (2002) [3].

$$wDIC = \frac{e^{-0.5(DIC_i - DIC_{min})}}{\sum_{i=1}^{n} e^{-0.5(DIC_i - DIC_{min})}} \quad (2)$$

After the selection of a random-effects structure, log-log PGLMM with the different combinations of fixed-effects terms were fitted, and a second phase of model selection was undertaken on the basis of DIC and *w*DIC [4].

Results

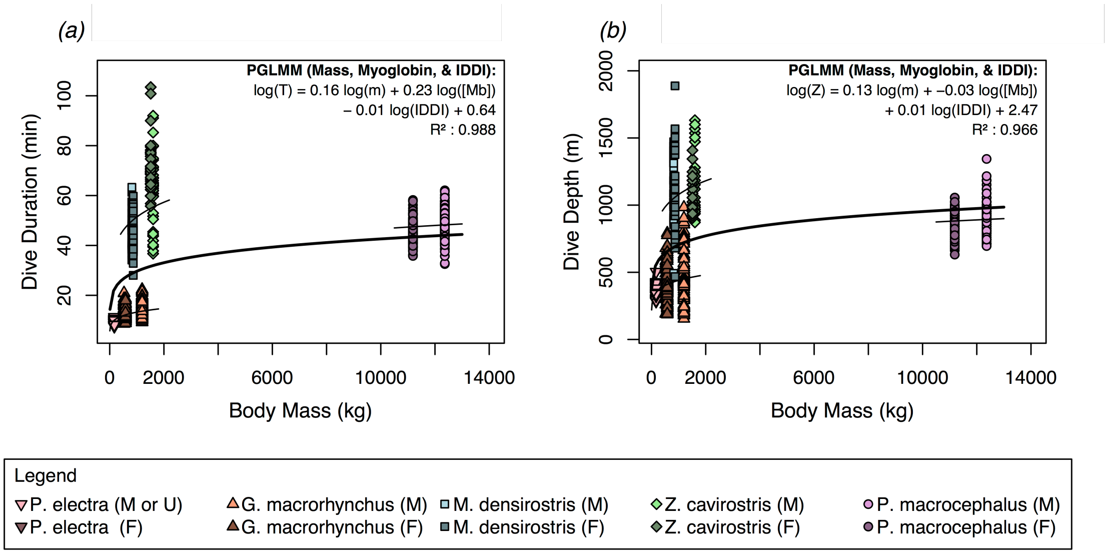

Figure A. Patterns of (a) dive duration (*T*) and (b) dive depth (*Z*) with respect to body mass (*m*) at the level of individual dives.

Overlaid on plots (a) and (b) are the predicted relationships from phylogenetic generalized linear mixed models (PGLMM) relating *T* and *Z* with *m*, myoglobin concentration ([*Mb*]), and inter-deep-dive interval (IDDI). The bold solid lines represent the main effect of the fixed effect parameters, while predictions including mean of random intercept parameters representing each family are shown in the stippled lines.

Fig A parts a and b illustrate that variation in the duration (*T*) and depth (*Z*) of individual dives was considerably greater than the variation in T_{max} among individuals (Fig 3). Fixed-effects-only GLM (Mod. 1d and 1f, Table A and B) that explained the variation in *T* and *Z* solely on the basis of covariates *m, Mb,* and *IDDI* proved inadequate. Instead, PGLMM that included random effects of individuals nested within phylogenetic correlations between species (Mod. 3d and 3f, Table A and B) were selected on the basis of wDIC values. The relatively low marginal coefficients of determination (R_m^2) for the PGLMM (Mod. 1-7e and Mod. 1-7g) indicated that the fixed-effects components of these models contributed minimally to the explanation of variation in *T* and *Z* after accounting for phylogenetic and random individual effects. This is illustrated by the large random-effects offsets of the delphinid, physeterid, and ziphiid families from the main effect of the fixed-effects covariates shown in Figure Aa and Ab, and by the relatively small coefficient of the log(IDDI) term when compared to the log(IDDI) coefficient in models

of T_{max}. Although not selected on the basis of wDIC, the R_m^2 values of 0.56 and 0.75 for the fixed-effects-only GLM models of *T* and *Z* (Mod. 1d and 1f) suggest that these fixed effects could also explain a substantial portion of the variance in both response variables. Overall, Figures 8a and 8b illustrate patterns similar to those observed in T_{max} also hold in T and Z.

Table A. Comparison generalized linear models and phylogenetic generalized linear mixed models of dive duration.

Models were developed at the level of individual dives, with fixed effect covariates including body mass, myoglobin concentration, and/or inter-deep-dive interval. The first section (above solid line) compares a fixed-effect only model with mixed model,s which includes random effects of individual and/or species within a phylogenetic correlation structure. The second section (below solid line) compares different combinations of the fixed effect covariates within the phylogenetic generalized linear mixed model structure selected in the first section. Marginal and conditional coefficients of determination indicate the proportion of variance explained by the fixed effects only and the full mixed effects model, respectively [5].

Model Formula	*k*	R_m^2	R_c^2	DIC	ΔDIC	*w*DIC
Mod. 1d (GLM):<br>log(*T*) ~ log(*m*) + log([*Mb*]) + log(*IDDI*)	4	0.75	-	-814.72	1426.44	0
Mod. 2d (PGLMM):<br>log(*T*) ~ log(*m*) + log([*Mb*]) + log(*IDDI*) | *SPP*	5	0.07	0.98	-2141.9	99.26	0
Mod. 3d (PGLMM):<br>log(*T*) ~ log(*m*) + log([*Mb*]) + log(*IDDI*) | *SPP* + *PTT*	6	0.04	0.99	-2241.16	0	1
Mod. 1e (PGLMM):<br>log(*T*) ~ log(*m*) + log([*Mb*]) + log(*IDDI*) | *SPP* + *PTT*	6	0.04	0.99	-2241.09	0.03	0.2
Mod. 2e (PGLMM):<br>log(*T*) ~ log(*m*) + log(*IDDI*) | *SPP* + *PTT*	5	0.10	0.98	-2241.08	0.04	0.2
Mod. 3e (PGLMM):<br>log(*T*) ~ log(*m*) + log([*Mb*]) | *SPP* + *PTT*	5	0.05	0.99	-2238.78	2.34	0.06
Mod. 4e (PGLMM):<br>log(*T*) ~ log(*IDDI*) + log([*Mb*]) | *SPP* + *PTT*	5	0.01	0.99	-2241.12	0	0.2
Mod. 5e (PGLMM):<br>log(*T*) ~ log(*m*) | *SPP* + *PTT*	4	0.11	0.98	-2238.75	2.37	0.06
Mod. 6e (PGLMM):<br>log(*T*) ~ log([*Mb*]) | *SPP* + *PTT*	4	0.01	0.99	-2238.8	2.32	0.06
Mod. 7e (PGLMM):<br>log(*T*) ~ log(*IDDI*) | *SPP* + *PTT*	4	<0.01	0.99	-2241.11	0.01	0.2

GLM, generalized linear models; PGLMM; phylogenetic generalized linear mixed models; *T*, dive duration; *SPP*, species; *m*, body mass; [*Mb*], myoglobin concentration; *IDDI*, inter-deep-dive interval; DIC, Deviance Information Criterion; ΔDIC, DIC difference; *w*DIC, DIC weights [3]; *k*, number of model parameters; R_m^2, marginal coefficient of determination [5]; R_c^2, conditional coefficient of determination [5].

Table B. Comparison of a generalized linear model and phylogenetic generalized linear mixed models of dive depth at the level of individual dives.
See details and interpretation of model selection metrics in Table A.

Model Formula	*k*	R_m^2	R_c^2	DIC	ΔDIC	*w*DIC
Mod. 1f (GLM): log(*Z*) ~ log(*m*) + log([*Mb*]) + log(*IDDI*)	4	0.56	-	-864.54	504.83	0
Mod. 2f (PGLMM): log(*Z*) ~ log(*m*) + log([*Mb*]) + log(*IDDI*) | *SPP*	5	0.06	0.91	-1343.58	25.79	0
Mod. 3f (PGLMM): log(*Z*) ~ log(*m*) + log([*Mb*]) + log(*IDDI*) | *SPP* + *PTT*	6	0.03	0.94	-1369.37	0	1
Mod. 1g (PGLMM): log(*Z*) ~ log(*m*) + log([*Mb*]) + log(*IDDI*) | *SPP* + *PTT*	6	0.02	0.97	-1369.33	1.31	0.1
Mod. 2g (PGLMM): log(*Z*) ~ log(*m*) + log(*IDDI*) | *SPP* + *PTT*	5	0.07	0.9	-1369.48	1.16	0.11
Mod. 3g (PGLMM): log(*Z*) ~ log(*m*) + log([*Mb*]) | *SPP* + *PTT*	5	0.04	0.93	-1370.54	0.1	0.19
Mod. 4g (PGLMM): log(*Z*) ~ log(*IDDI*) + log([*Mb*]) | *SPP* + *PTT*	5	0	0.93	-1369.44	1.2	0.11
Mod. 5g (PGLMM): log(*Z*) ~ log(*m*) | *SPP* + *PTT*	4	0.07	0.9	-1370.64	0	0.2
Mod. 6g (PGLMM): log(*Z*) ~ log([*Mb*]) | *SPP* + *PTT*	4	0	0.93	-1370.6	0.04	0.19
Mod. 7g (PGLMM): log(*Z*) ~ log(*IDDI*) | *SPP* + *PTT*	4	0	0.91	-1369.46	1.18	0.11

GLM, generalized linear models; PGLMM; phylogenetic generalized linear mixed models; *Z*, dive depth; *SPP*, species; *m*, body mass; [*Mb*], myoglobin concentration; *IDDI*, inter-deep-dive interval; DIC, Deviance Information Criterion; ΔDIC, DIC difference; *w*DIC, DIC weights [3]; *k*, number of model parameters; R_m^2, marginal coefficient of determination [5]; R_c^2, conditional coefficient of determination [5].

References

1. de Villemereuil P, Nakagawa S. General quantitative genetic methods for comparative biology. In: Garamszegi LZ, editor. Modern Phylogenetic Comparative Methods and Their Application in Evolutionary Biology. Berlin: Springer; 2014. pp. 287–303.

2. McGowen MR, Spaulding M, Gatesy J. Divergence date estimation and a comprehensive molecular tree of extant cetaceans. Mol Phylogenet Evol. 2009;53: 891–906.

3. Burnham KP, Anderson DR. Model Selection and Multimodel Inference. New York: Springer New York; 2004.

4. Zuur AF, Ieno EN, Walker NJ, Saveliev AA, Smith GM. Mixed effects models and extensions in ecology with R. New York: Springer New York; 2009.

5. Nakagawa S, Schielzeth H. A general and simple method for obtaining R^2 from generalized linear mixed-effects models. Methods Ecol Evol. 2013;4: 133–142.
